# Prevalence of 12 Common Health Conditions in Sexual and Gender Minority Participants in the All of Us Research Program

**DOI:** 10.1001/jamanetworkopen.2023.24969

**Published:** 2023-07-31

**Authors:** Nguyen K. Tran, Mitchell R. Lunn, Claire E. Schulkey, Samantha Tesfaye, Siddhartha Nambiar, Snigdhansu Chatterjee, Dawn Kozlowski, Paula Lozano, Fornessa T. Randal, Yicklun Mo, Siya Qi, Ell Hundertmark, Chloe Eastburn, Anthony T. Pho, Zubin Dastur, Micah E. Lubensky, Annesa Flentje, Juno Obedin-Maliver

**Affiliations:** 1 The PRIDE Study/PRIDEnet, Departments of Medicine and of Obstetrics and Gynecology, Stanford University School of Medicine, Palo Alto, California; 2 Division of Nephrology, Department of Medicine, Stanford University School of Medicine, Stanford, California; 3 Department of Epidemiology and Population Health, Stanford University School of Medicine, Stanford, California; 4 All of Us Research Program, Office of the Director, National Institutes of Health, Bethesda, Maryland; 5 Covalent Solutions LLC, Rockville, Maryland; 6 School of Statistics, University of Minnesota, Minneapolis; 7 Cherokee Health Systems, Knoxville, Tennessee; 8 Center for Asian Health Equity, The University of Chicago Medicine, Chicago, Illinois; 9 Asian Health Coalition, Chicago, Illinois; 10 Division of Gynecology and Gynecologic Specialties, Department of Obstetrics and Gynecology, Stanford University School of Medicine, Stanford, California; 11 Los Angeles LGBT Center, Los Angeles, California; 12 Department of Community Health Systems, University of California, San Francisco; 13 Department of Psychiatry and Behavioral Sciences, University of California, San Francisco

## Abstract

**Importance:**

Limited data describe the health status of sexual or gender minority (SGM) people due to inaccurate and inconsistent ascertainment of gender identity, sex assigned at birth, and sexual orientation.

**Objective:**

To evaluate whether the prevalence of 12 health conditions is higher among SGM adults in the All of Us Research Program data compared with cisgender heterosexual (non-SGM) people.

**Design, Setting, and Participants:**

This cross-sectional study used data from a multidisciplinary research consortium, the All of Us Research Program, that links participant-reported survey information to electronic health records (EHR) and physical measurements. In total, 372 082 US adults recruited and enrolled at an All of Us health care provider organization or by directly visiting the enrollment website from May 31, 2017, to January 1, 2022, and were assessed for study eligibility.

**Exposures:**

Self-identified gender identity and sexual orientation group.

**Main Outcomes and Measures:**

Twelve health conditions were evaluated: 11 using EHR data and 1, body mass index (BMI; calculated as weight in kilograms divided by height in meters squared), using participants’ physical measurements. Logistic regression (adjusting for age, income, and employment, enrollment year, and US Census division) was used to obtain adjusted odds ratios (AORs) for the associations between each SGM group and health condition compared with a non-SGM reference group.

**Results:**

The analytic sample included 346 868 participants (median [IQR] age, 55 [39-68] years; 30 763 [8.9%] self-identified as SGM). Among participants with available BMI (80.2%) and EHR data (69.4%), SGM groups had higher odds of anxiety, depression, HIV diagnosis, and tobacco use disorder but lower odds of cardiovascular disease, kidney disease, diabetes, and hypertension. Estimated associations for asthma (AOR, 0.39 [95% CI, 0.24-0.63] for gender diverse people assigned male at birth; AOR, 0.51 [95% CI, 0.38-0.69] for transgender women), a BMI of 25 or higher (AOR, 1.65 [95% CI, 1.38-1.96] for transgender men), cancer (AOR, 1.15 [95% CI, 1.07-1.23] for cisgender sexual minority men; AOR, 0.88 [95% CI, 0.81-0.95] for cisgender sexual minority women), and substance use disorder (AOR, 0.35 [95% CI, 0.24-0.52] for gender diverse people assigned female at birth; AOR, 0.65 [95% CI, 0.49-0.87] for transgender men) varied substantially across SGM groups compared with non-SGM groups.

**Conclusions and Relevance:**

In this cross-sectional analysis of data from the All of Us Research Program, SGM participants experienced health inequities that varied by group and condition. The All of Us Research Program can be a valuable resource for conducting health research focused on SGM people.

## Key Points

**Question:**

Is the prevalence of certain health conditions higher among sexual and gender minority (SGM) people in the US compared with cisgender and heterosexual (non-SGM) people?

**Findings:**

In this cross-sectional study of 30 763 SGM and 316 105 non-SGM adults enrolled in the All of Us Research Program, the odds of 8 health conditions were significantly higher in at least 1 of 6 SGM groups compared with their non-SGM counterparts.

**Meaning:**

The findings of this study suggest that there are disparities for certain health conditions among SGM adults compared with their non-SGM counterparts.

## Teaser

This cross-sectional study uses data from the All of Us Research Program to assess whether the prevalence of 12 common health conditions differs between sexual and gender minority adults and cisgender heterosexual adults.

## Introduction

Sexual and gender minority (SGM) people, including individuals who identify as asexual, bisexual, gay, intersex, lesbian, nonbinary, queer, transgender, or two-spirit, represent a growing population in the US. Although estimates vary, approximately 7.2% of US adults identify as lesbian, gay, bisexual, transgender, and queer or questioning.^1^ Due to stigma, discrimination, and minority stress (ie, prejudicial events, expectation of rejection, identity concealment, or internalized homophobia), SGM people often experience greater health and health care inequities relative to their cisgender heterosexual counterparts.^2,3,4,5^ (Cisgender refers to individuals whose gender identity aligns with the gender commonly associated with the sex assigned to them at birth.) These health inequities include difficulties accessing equitable and culturally competent health care services, chronic health conditions, infectious diseases, mental health, and substance use.^6,7,8,9,10,11,12,13,14,15,16,17,18,19,20,21,22,23,24,25,26,27,28^ The National Institutes of Health has recognized SGM people as a “health disparity population for research.”^29^ However, resolving these inequities is hindered by data quality issues, such as inadequate sample sizes and limited social and biomedical data.^30,31^ These limitations can bias research findings,^32^ prevent data disaggregation of SGM populations to evaluate social and clinical exposures on health outcomes (eg, transgender people, gender diverse people, and minoritized racial or ethnic groups), and may negatively affect SGM health care practices.

There are compelling needs for larger and more diverse SGM samples that integrate multiple data sources to identify health inequities and inform treatments and policies for intervention. Prior research has relied on small convenience samples, but studies have increasingly used population-based surveys^6,7,8,9,11,13,16,17,21^ and electronic health records (EHRs) to examine health disparities among SGM populations.^18,33,34^ Although population-based surveys are often used to produce weighted estimates representative of a target population,^35,36^ EHR data also have the potential to improve health care quality for SGM populations. Recent developments in methods to identify transgender people in claims data, in the absence of gender identity data, have provided promising results.^37,38^ However, such data sources are flawed for examining SGM health inequities due to their inaccurate and inconsistent ascertainment of gender identity, sex assigned at birth, and sexual orientation.^30,31^ To increase overall participation and meet specific community health needs, scholars and advocates have called for community-engaged research to forge relationships with populations underrepresented in biomedical research.^39,40^ However, community-engaged research often lacks large volumes of diverse participants and comprehensive EHR data. Integrating data across sources to improve data quality could advance prevention and treatment of disease for SGM populations.

The National Institutes of Health’s All of Us Research Program addresses these current challenges. It is a national, community-engaged program that aims to improve health and health care practices by partnering with 1 million volunteer participants, mostly from communities historically underrepresented in biomedical research across the US.^41^ The program links participant-reported information (including sexual orientation, gender identity, and sex assigned at birth) with EHR data, biospecimens, physical measurements, genomic data, and digital health technologies, cleaning, sorting, and unifying these disparate data to create a single repository (ie, data harmonization). With the large volume of All of Us data from different sources, demonstration projects are necessary to describe the cohort and illustrate the data quality for future health research.^42^ Therefore, this study aims to evaluate the potential of the All of Us data for SGM health disparities research by describing the sociodemographic and health conditions of SGM people compared with non-SGM people.

## Methods

### Data Sources and Participants

We conducted a cross-sectional analysis using the controlled tier v6 curated data repository (C2022Q2R2) of the All of Us Research Program.^41^ Data were collected for US rfesidents aged 18 years or older who enrolled from May 31, 2017, to January 1, 2022, through a health care provider organization or the enrollment website. Available data sources were harmonized using the Observational Medical Outcomes Partnership Common Data Model (version 5.2) by the All of Us Data and Research Center.^41^ Data sources included health surveys administered in English or Spanish, physical measurements, and EHR-documented health conditions (eTable 1 in Supplement 1). This study was approved by the All of Us Research Program Science Committee, and the requirement for informed consent was waived by the All of Us Institutional Review Board because it was considered nonhuman participants research. This study followed the Strengthening the Reporting of Observational Studies in Epidemiology (STROBE) guideline for cross-sectional studies.^43^

**Figure 1.**
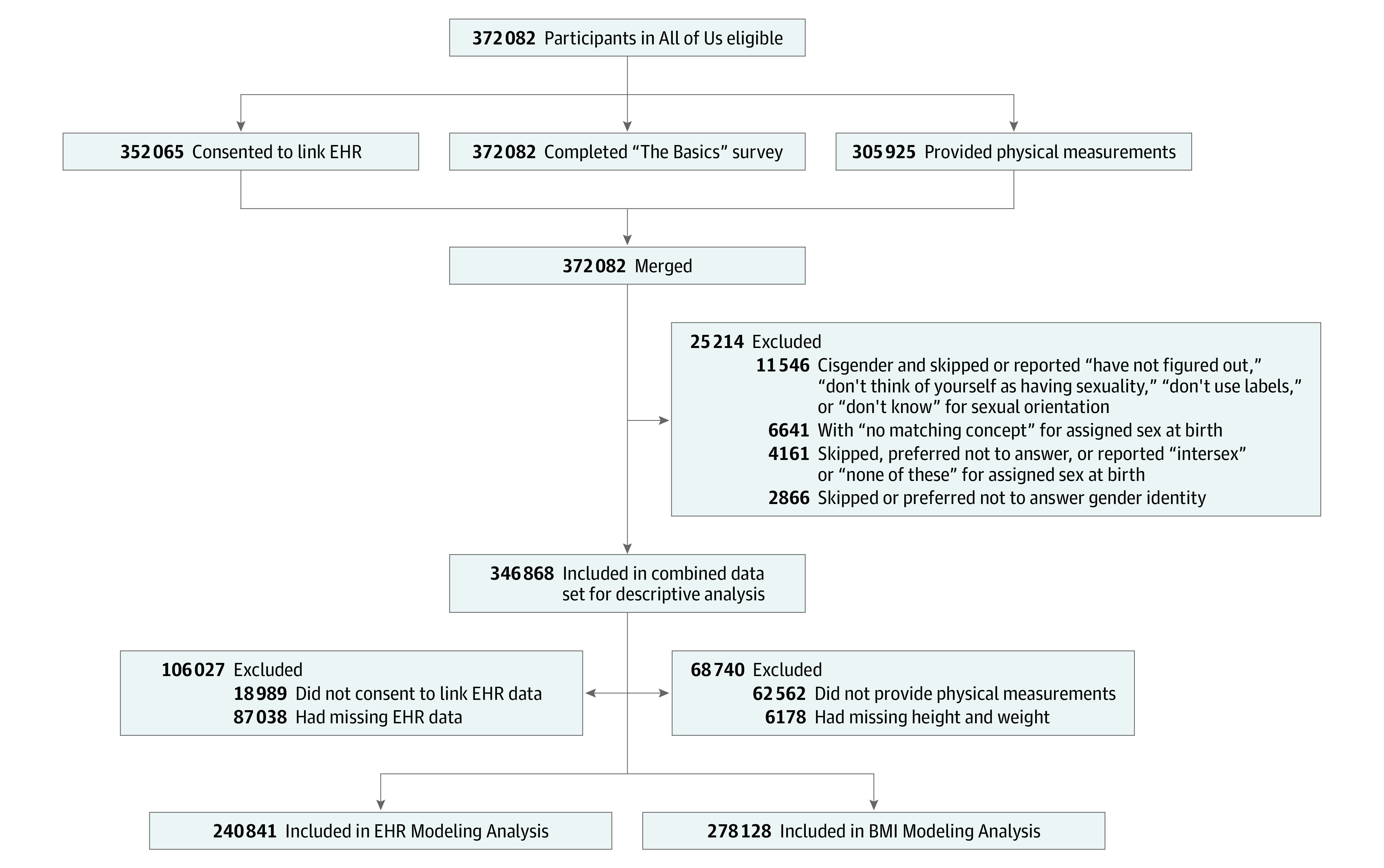
Participant Flowchart No matching concept refers to missing data in the All of Us curated data repository due to a technological error that resulted in previous selections being overwritten with null values (or the participant skipped the item). BMI indicates body mass index; EHR, electronic health record.

Descriptive analysis excluded some of 372 082 participants eligible in All of Us for the following reasons (Figure 1): (1) a “no matching concept” value for sex assigned at birth indicating missing data due to a technological error that resulted in previous selections being overwritten with null values or that the participant skipped the item; (2) participants skipping sex assigned at birth, preferring not to answer, or reporting “intersex” or “none of these”; (3) participants skipping gender identity or preferring not to answer; and (4) cisgender participants skipping sexual orientation or reporting “have not figured out,” “don’t think of yourself as having sexuality,” “don’t use labels,” or “don’t know”. Data were collected on intersex participants but excluded from this analysis due to small sample size and privacy concerns.

### Measures

#### Sexual Orientation– or Gender Identity–Based Groups

Participants provided their self-identified gender identity, sex assigned at birth, and sexual orientation, and their responses were categorized into 8 mutually exclusive categories (eTable 2 in Supplement 1): (1) cisgender heterosexual men, (2) cisgender heterosexual women, (3) cisgender sexual minority men, (4) cisgender sexual minority women, (5) gender diverse (ie, outside of the man or woman binary) people assigned female sex at birth (AFAB) of any sexual orientation, (6) gender diverse people assigned male sex at birth (AMAB) of any sexual orientation, (7) transgender men of any sexual orientation, and (8) transgender women of any sexual orientation. These categories were selected to highlight the diversity of SGM populations and encompass groups affected by cisheterosexism, a sociostructural system that privileges cisgender heterosexual identities at the expense of marginalizing SGM individuals.

#### Patient-Reported Outcome Measures

We assessed physical and mental health using 2 subscales from the Patient-Reported Outcomes Measurement Information System (PROMIS) Global Health survey (version 1.0).^44^ The PROMIS physical health (PROMIS-PH) subscale consists of 4 items on physical health, physical functioning, pain, and fatigue, and the PROMIS mental health (PROMIS-MH) subscale includes 4 items on mental health, emotional problems, satisfaction with discretionary social activities, and quality of life.^44^ Responses, aside from pain, were based on a 5-point Likert scale (range, 1-5), with higher values representing better health. Pain was scored from 0 to 10 but was rescored to a 5-point scale as recommended by Hays et al^44^ (0 = 1; 1-3 = 2; 4-6 = 3; 7-9 = 4; 10 = 5, with higher values representing worse pain). Raw scores were calculated for each subscale (range, 4-20) and standardized to *T* score values with a mean of 50 and standard deviation of 10.^45^ PROMIS-PH scores less than 42 and PROMIS-MH scores less than 40 were used as established *T* score cutoffs for poor or fair health ratings.^46^

#### Substance Use

Participants’ substance use was assessed using 3 measures: the Alcohol Use Disorders Identification Test-Concise (AUDIT-C) scale, questions about any substance use in the previous 3 months (eg, cannabis, cocaine, stimulants, inhalants, sedatives, hallucinogens, and opioids), and current tobacco smoking status. The AUDIT-C scale contains 3 items: frequency of alcohol use, number of daily alcohol drinks, and frequency of binge drinking,^47^ with responses on a 5-point Likert scale (range, 0-4) and scores calculated by summing across items (range, 0-12). Past-year hazardous alcohol consumption was determined based on established cutoffs by gender groups (women, ≥3; men, ≥4),^48^ and both cutoffs were reported for gender diverse participants.

#### Health Conditions

We calculated body mass index (BMI; calculated as weight in kilograms divided by height in meters squared). A BMI of 25.0 or higher was considered overweight or obese. We extracted Systematized Nomenclature of Medicine (SNOMED) codes for 11 additional health conditions broadly defined as anxiety, asthma, cancer, cardiovascular disease (ie, myocardial infarction, stroke, and coronary artery disease), chronic kidney disease, depression, diabetes, HIV diagnosis, hypertension, substance use, and tobacco use (eTable 1 in Supplement 1). For each health condition, we required a SNOMED code to be reported on at least 2 different dates to indicate the presence of the condition.

#### Covariates

Our analysis accounted for age, household income, employment, enrollment year, and US census division in adjusted models. In addition to gender identity, sex assigned at birth, and sexual orientation, we included data on self-reported racial and/or ethnic identity (African American or Black, Asian, Hispanic or Latinx, Middle Eastern or North African, Native Hawaiian or Other Pacific Islander, or White), educational level, homeownership, and health insurance status to describe the sample.

### Statistical Analysis

Data were analyzed from November 20, 2022, to May 23, 2023. We evaluated differences in key characteristics between SGM and non-SGM groups. Using logistic regression, we conducted a complete case analysis among participants with available physical measurements and EHR data by comparing differences for each health condition between cisgender sexual minority groups and groups of gender minority participants of any sexual orientation with their cisgender heterosexual counterparts. Gender diverse participants were compared with cisgender heterosexual men and cisgender heterosexual women due to limited data on how cisheterosexism perpetuates health inequities for gender diverse individuals when compared with cisgender heterosexual counterparts. We pursued a complete case analysis because the overall focus of demonstration projects is to describe the utility of All of Us data, but we also evaluated the proportion of missingness in the overall sample and among participants with BMI and EHR data to understand how it may have biased our results (eTable 3 in Supplement 1). For sociodemographic, PROMIS, AUDIT-C, and the self-reported substance use and smoking status, we treated missingness as a separate category, indicating nonresponse due to skipping or preferring not to answer the survey question.

We conducted a post hoc analysis to assess whether the robustness of our findings was modified by residual confounding. We used inverse probability weights to generate a pseudopopulation that balanced measured covariates between SGM groups and their non-SGM counterparts. We estimated propensity scores using logistic regression and assessed conditional exchangeability through density plots (eFigure 2 in Supplement 1). To ensure covariate balance, we evaluated standardized mean differences (eFigures 3 and 4 in Supplement 1). We then refitted our models to estimate the propensity score–weighted odds ratios (ORs).

All analyses were conducted in the All of Us Researcher Workbench (a cloud-based analytic platform) in R, version 4.2,^49^ using the WeightIt package to estimate the weights.^50^ We reported adjusted ORs (AORs) and 95% CIs for the primary findings. Statistical significance was defined as a 95% CI excluding 1. Results followed the All of Us Data and Statistics Dissemination Policy disallowing disclosure of cell counts from 1 to 19.^51^

## Results

Of 372 082 potential participants, 346 868 (93.2%) included in the descriptive analysis (Figure 1) had a median (IQR) age of 55 years (39-68) years; 70 755 (20.4%) were African American or Black, 14 237 (4.1%) were Asian, 62 357 (18.0%) were Hispanic or Latinx, 3647 (1.1%) were Middle Eastern or North African, 880 (0.3%) were Native Hawaiian or Other Pacific Islander, and 201 128 (58.0%) were White; 30 763 (8.9%) identified as SGM; and 316 105 (91.1%) identified as non-SGM (eTable 3 in Supplement 1). In the SGM group, 10 980 participants (35.7%) were cisgender sexual minority men, 16 096 participants (52.3%) were cisgender sexual minority women, 1433 participants (4.7%) were gender diverse people AFAB, 482 participants (1.6%) were gender diverse people AMAB, 923 participants (3.0%) were transgender men, and 849 participants (2.8%) were transgender women (Table 1). Overall, SGM groups were younger (median age range, 32-51 years for 6 SGM groups vs 55-59 years for 2 non-SGM groups), had a higher proportion of individuals with median household incomes less than $25 000 annually (range, 31.7%-43.4% for 6 SGM groups vs 24.2%-26.6% for 2 non-SGM groups), and a lower median percentage of homeownership (range, 20.4%-33.3% for 6 SGM groups vs 46.7%-48.3% for 2 non-SGM groups). However, differences in minoritized racial and/or ethnic identity, educational level, and employment varied considerably across SGM and non-SGM groups. More detailed information on sociodemographic characteristics, PROMIS and AUDIT-C scores, substance use, and tobacco smoking is given in eTable 3 in Supplement 1 for the analytic sample and by SGM group in Table 1.

**Table 1.** Sociodemographic Characteristics, Physical and Mental Health, and Self-Reported Substance Use of 346 950 Participants in the All of Us Research Program by Sexual Orientation and Gender Identity Groups

Characteristic	Participants, No. (%)							
	Cisgender heterosexual men	Cisgender heterosexual women	Cisgender sexual minority men	Cisgender sexual minority women	Gender diverse people AFAB	Gender diverse people AMAB	Transgender men	Transgender women
Total No. of participants	120 568	195 537	10 980	16 096	1433	482	923	849
Age, mean (IQR), y	59 (43-70)	55 (39-67)	51 (36-63)	38 (30-53)	32 (26-41)	36 (29-50)	40 (30-55)	48 (35-62)
Race and ethnicity^a^^,^^b^								
African American or Black	26 705 (22.2)	38 110 (19.5)	2180 (19.9)	3181 (19.8)	124 (8.7)	53 (11.0)	190 (20.6)	212 (25.0)
Asian	4927 (4.1)	7964 (4.1)	472 (4.3)	684 (4.3)	95 (6.6)	36 (7.5)	35 (3.8)	24 (2.8)
Hispanic or Latinx	18 383 (15.3)	38 931 (19.9)	1724 (15.7)	25,75 (16.0)	160 (11.2)	56 (11.6)	321 (34.8)	213 (25.1)
Middle Eastern or North African	1401 (1.2)	1865 (1.0)	139 (1.3)	170 (1.1)	33 (2.3)	<20	<20	<20
Native Hawaiian or Other Pacific Islander	312 (0.3)	461 (0.2)	36 (0.3)	56 (0.3)	<20	0	<20	<20
White	69 674 (57.8)	111 942 (57.3)	6820 (62.0)	10 401 (64.6)	1151 (80.3)	344 (71.4)	390 (42.3)	406 (47.8)
Sexual orientation^a^^,^^b^								
Asexual	0	0	82 (0.8)	323 (2.0)	104 (7.3)	<20	<20	<20
Bisexual	0	0	2804 (25.5)	9636 (59.9)	409 (28.5)	117 (24.3)	135 (14.6)	141 (16.6)
Gay	0	0	7587 (69.1)	484 (3.0)	56 (3.9)	109 (22.6)	73 (7.9)	84 (9.9)
Lesbian	0	0	39 (0.4)	4240 (26.3)	211 (14.7)	<20	48 (5.2)	98 (11.5)
Mostly straight	0	0	156 (1.4)	506 (3.1)	<20	<20	<20	<20
Queer	0	0	42 (0.4)	311 (1.9)	289 (20.2)	56 (11.6)	48 (5.2)	<20
Polysexual, omnisexual, sapiosexual, or pansexual	0	0	81 (0.7)	408 (2.5)	149 (10.4)	42 (8.7)	29 (3.1)	24 (2.8)
Straight	120 568 (100)	195 537 (100)	225 (2.1)	695 (4.3)	109 (7.6)	93 (19.3)	455 (49.3)	355 (41.8)
Two-spirit	0	0	33 (0.3)	<20	<20	<20	<20	<20
Gender identity^a^								
Genderfluid, genderqueer, gender variant, unsure, specific gender, or two spirit	0	0	0	0	169 (11.8)	87 (18.1)	0	0
Man	120 568 (100)	0	10 980 (100)	0	0	0	486 (52.7)	0
Nonbinary	0	0	0	0	1236 (86.3)	380 (78.8)	0	0
Transgender	0	0	0	0	244 (17.0)	64 (13.3)	353 (38.2)	418 (49.2)
Woman	0	195 537 (100)	0	16 096 (100)	0	0	0	386 (45.5)
Annual household income, $								
<25 000	32 028 (26.6)	47 316 (24.2)	3479 (31.7)	5349 (33.2)	472 (32.9)	173 (35.9)	383 (41.5)	368 (43.4)
25 000-49 999	15 783 (13.1)	31 450 (16.1)	1926 (17.5)	3060 (19.0)	330 (23.0)	96 (19.9)	131 (14.2)	117 (13.8)
50 000-99 999	21 587 (17.9)	38 580 (19.7)	2099 (19.1)	3067 (19.1)	289 (20.2)	78 (16.2)	107 (11.6)	85 (10.0)
100 000-149 999	12 795 (10.6)	19 732 (10.1)	1075 (9.8)	1421 (8.8)	107 (7.5)	48 (10.0)	40 (4.3)	44 (5.2)
≥150 000	15 788 (13.1)	20 461 (10.5)	1204 (11.0)	1328 (8.3)	94 (6.6)	40 (8.3)	40 (4.3)	36 (4.2)
Prefer to not answer or skipped	22 587 (18.7)	37 998 (19.4)	1197 (11.0)	1871 (11.6)	141 (9.8)	47 (9.8)	222 (24.1)	199 (23.4)
Some college or higher	79 708 (66.1)	141 414 (72.3)	8438 (76.8)	11 753 (73.0)	1219 (85.1)	374 (77.6)	489 (53.0)	421 (49.6)
Employed for wages	43 823 (36.4)	88 547 (45.3)	4977 (45.3)	8248 (51.2)	838 (58.5)	238 (49.4)	362 (39.2)	290 (34.2)
Own a home	56 271 (46.7)	94 365 (48.3)	3656 (33.3)	4745 (29.5)	320 (22.3)	118 (24.5)	188 (20.4)	190 (22.4)
Health insurance	106 726 (88.5)	181 527 (92.8)	9974 (90.8)	14 704 (91.4)	1345 (93.9)	439 (91.1)	757 (82.0)	718 (84.6)
PROMIS^c^								
GPH poor or fair	21 039 (17.5)	41 305 (21.1)	1812 (16.5)	4007 (24.9)	407 (28.4)	89 (18.5)	241 (26.1)	212 (25.0)
GMH poor or fair	15 641 (13.0)	26 590 (13.6)	2196 (20.0)	4338 (27.0)	606 (42.3)	172 (35.7)	253 (27.4)	237 (27.9)
AUDIT-C score^d^								
≥3	ND	58 082 (29.7)	ND	6466 (40.2)	492 (34.3)	217 (45.0)	ND	278 (32.7)
≥4	38 187 (31.7)	ND	3817 (34.8)	ND	306 (21.4)	167 (34.7)	169 (18.3)	ND
Substance use in previous 3 mo^e^	11 655 (9.7)	14 489 (7.4)	1300 (11.8)	2339 (14.5)	241 (16.8)	61 (12.7)	116 (12.6)	112 (13.2)
Current smoker	26 670 (22.1)	23 052 (11.8)	2492 (22.7)	3418 (21.2)	140 (9.8)	93 (19.3)	176 (19.1)	219 (25.8)
Enrollment year								
2017-2018	34 848 (28.9)	60 502 (30.9)	3411 (31.1)	4652 (28.9)	322 (22.5)	123 (25.5)	250 (27.1)	227 (26.7)
2019	55 040 (45.7)	86 611 (44.3)	4602 (41.9)	6519 (40.5)	521 (36.4)	182 (37.8)	471 (51.0)	406 (47.8)
2020	16 994 (14.1)	25 753 (13.2)	1502 (13.7)	2234 (13.9)	220 (15.4)	69 (14.3)	108 (11.7)	103 (12.1)
2021-2022	13 686 (11.4)	22 671 (11.6)	1465 (13.3)	2691 (16.7)	370 (25.8)	108 (22.4)	94 (10.2)	113 (13.3)

Abbreviations: AFAB, assigned female at birth; AMAB, assigned male at birth; AUDIT-C, Alcohol Use Disorders Identification Test-Concise; ND, not determined; PROMIS, Patient-Reported Outcomes Measurement Information System; GMH, global mental health; GPH, global physical health.

^a^ Categories are not mutually exclusive; they do not sum to the column total because participants may self-identify in multiple groups.

^b^ Groups with 1 to 19 participants were expressed as having fewer than 20 in accordance with All of Us policy.

^c^ Scores of less than 40 for GPH and less than 42 for GMH were considered poor or fair.

^d^ Contains 3 items: frequency of alcohol use, number of daily alcohol drinks, and frequency of binge drinking,^47^ with responses on a 5-point Likert scale (range, 0-4) and scores calculated by summing across items (range, 0-12). Scores of 3 or greater were used as the cutoff for women, scores of 4 or greater were used as the cutoff for men, and both cutoffs were used for gender diverse people; cutoffs indicated screening positive for past-year hazardous alcohol consumption.

^e^ Included cannabis, cocaine, prescription and nonprescription stimulants, inhalants, sedatives, hallucinogens, or prescription and nonprescription opioids.

Measurement availability differed with BMI measurements for 278 128 participants (80.2%) and with EHR data for 240 841 participants (69.4%), with gender diverse people AFAB having the highest percentage of missing BMI (42.6%) and EHR (50.6%) data (eFigure 1 in Supplement 1). Among participants with available BMI and EHR data, compared with non-SGM groups, SGM groups had a higher prevalence of anxiety, depression, and HIV diagnosis but a lower prevalence of cancer, cardiovascular disease, kidney disease, diabetes, and hypertension (Table 2). Prevalence of asthma, BMI 25 or higher, substance use disorder, and tobacco use disorder varied more substantially across SGM and non-SGM groups. Unadjusted ORs of these health conditions are presented in eTables 4 to 11 in Supplement 1.

**Table 2.** Health Conditions of All of Us Research Program Participants by Sexual Orientation and Gender Identity Group

Condition	Participants, No. (%)							
	Cisgender heterosexual men	Cisgender heterosexual women	Cisgender sexual minority men	Cisgender sexual minority women	Gender diverse people AFAB	Gender diverse people AMAB	Transgender men	Transgender women
EHR condition (n = 240 841), No.^a^	83 890	137 593	7183	10 028	707	287	573	580
Anxiety	12 195 (14.5)	30 745 (22.3)	1626 (22.6)	2946 (29.4)	251 (35.5)	79 (27.6)	150 (26.2)	123 (21.2)
Asthma	6288 (7.5)	18 827 (13.7)	656 (9.1)	1694 (16.9)	119 (16.8)	<20	76 (13.3)	45 (7.8)
Cancer	12 254 (14.6)	17 119 (12.4)	831 (11.6)	665 (6.6)	29 (4.1)	20 (7.0)	38 (6.6)	43 (7.4)
Cardiovascular disease	13 246 (15.8)	11 116 (8.1)	798 (11.1)	450 (4.5)	23 (3.3)	<20	36 (6.3)	47 (8.1)
Chronic kidney disease	7417 (8.8)	7318 (5.3)	507 (7.1)	271 (2.7)	<20	<20	30 (5.2)	33 (5.7)
Depression	12 398 (14.8)	30 310 (22.0)	1790 (24.9)	2883 (28.8)	249 (35.2)	74 (25.8)	157 (27.4)	137 (23.6)
Diabetes	13 810 (16.5)	20 393 (14.8)	909 (12.7)	1018 (10.2)	46 (6.5)	22 (7.7)	83 (14.5)	82 (14.1)
HIV diagnosis	893 (1.1)	1015 (0.7)	1387 (19.3)	101 (1.0)	<20	<20	<20	43 (7.4)
Hypertension	31 240 (37.2)	44 444 (32.3)	2129 (29.6)	2046 (20.4)	99 (14.0)	45 (15.7)	147 (25.7)	168 (29.0)
Substance use disorder	7286 (8.7)	4719 (3.4)	752 (10.5)	793 (7.9)	27 (3.8)	23 (8.0)	49 (8.6)	57 (9.8)
Tobacco use disorder	4732 (5.6)	6233 (4.5)	497 (6.9)	658 (6.6)	27 (3.8)	<20	34 (5.9)	40 (6.9)
BMI recorded (n = 278 128), No.	100 398	155 364	8350	11 462	804	332	715	703
≥25	73 063 (72.8)	110 994 (71.4)	5585 (66.9)	8001 (69.8)	534 (66.4)	188 (56.6)	551 (77.1)	488 (69.4)

Abbreviations: AFAB, assigned female at birth; AMAB, assigned male at birth; BMI, body mass index (calculated as weight in kilograms divided by height in meters squared); EHR, electronic health record.

^a^ Groups with 1 to 19 participants were expressed as having fewer than 20 in accordance with All of Us policy.

**Figure 2.**
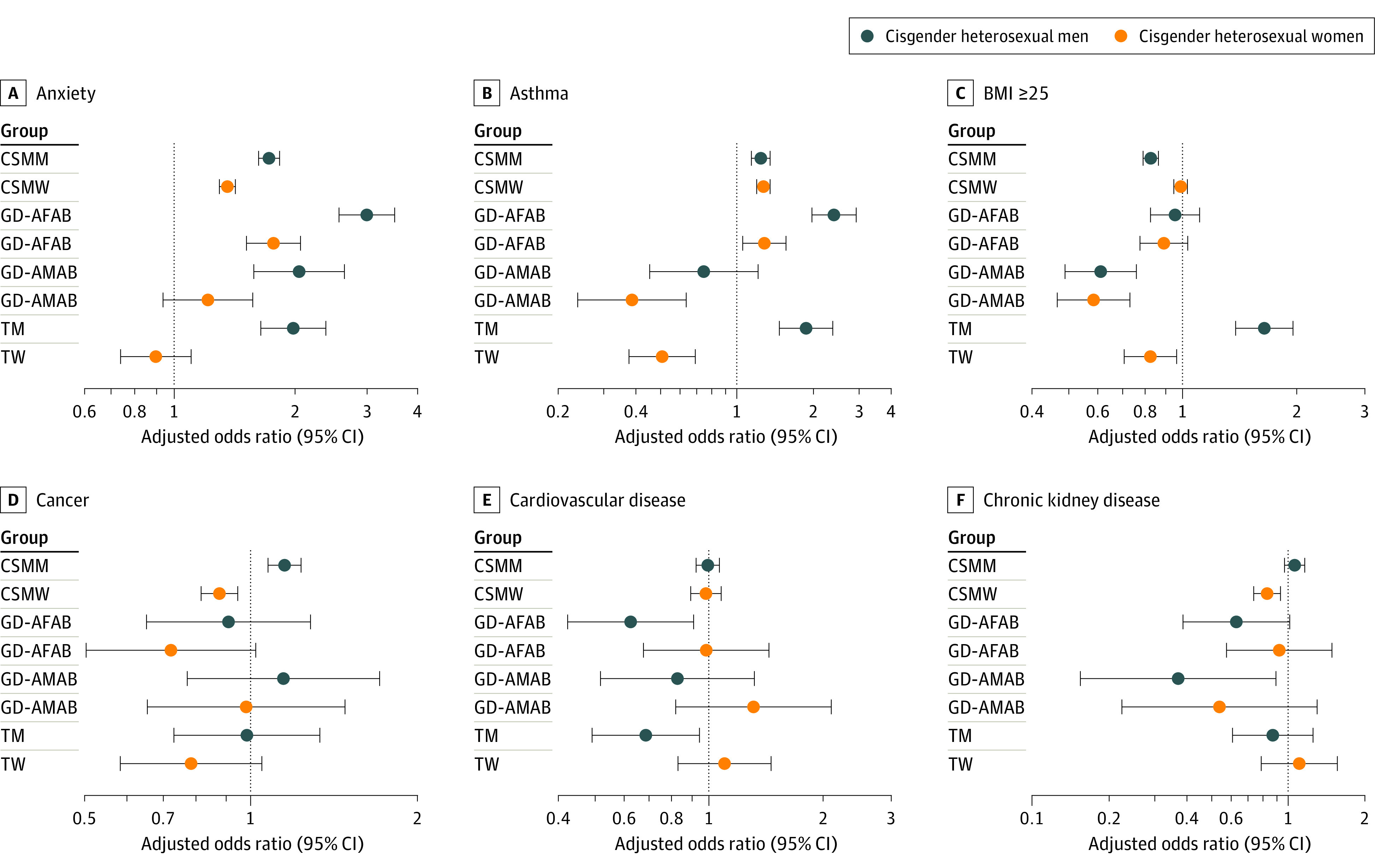
Adjusted Odds Ratios of Anxiety, Asthma, Body Mass Index (BMI) of 25 or Higher, Cancer, Cardiovascular Disease, and Chronic Kidney Disease Among Sexual and Gender Minority Participants in the All of Us Research Program, by Comparison Group Models controlled for current age (continuous), annual income, employment status, enrollment year, and US census division. BMI was calculated as weight in kilograms divided by height in meters squared; CSMM indicates cisgender sexual minority men; CSMW, cisgender sexual minority women; GD-AFAB, gender diverse people assigned female at birth of any sexual orientation; GD-AMAB, gender diverse people assigned male at birth of any sexual orientation; TM, transgender men of any sexual orientation; and TW, transgender women of any sexual orientation.

**Figure 3.**
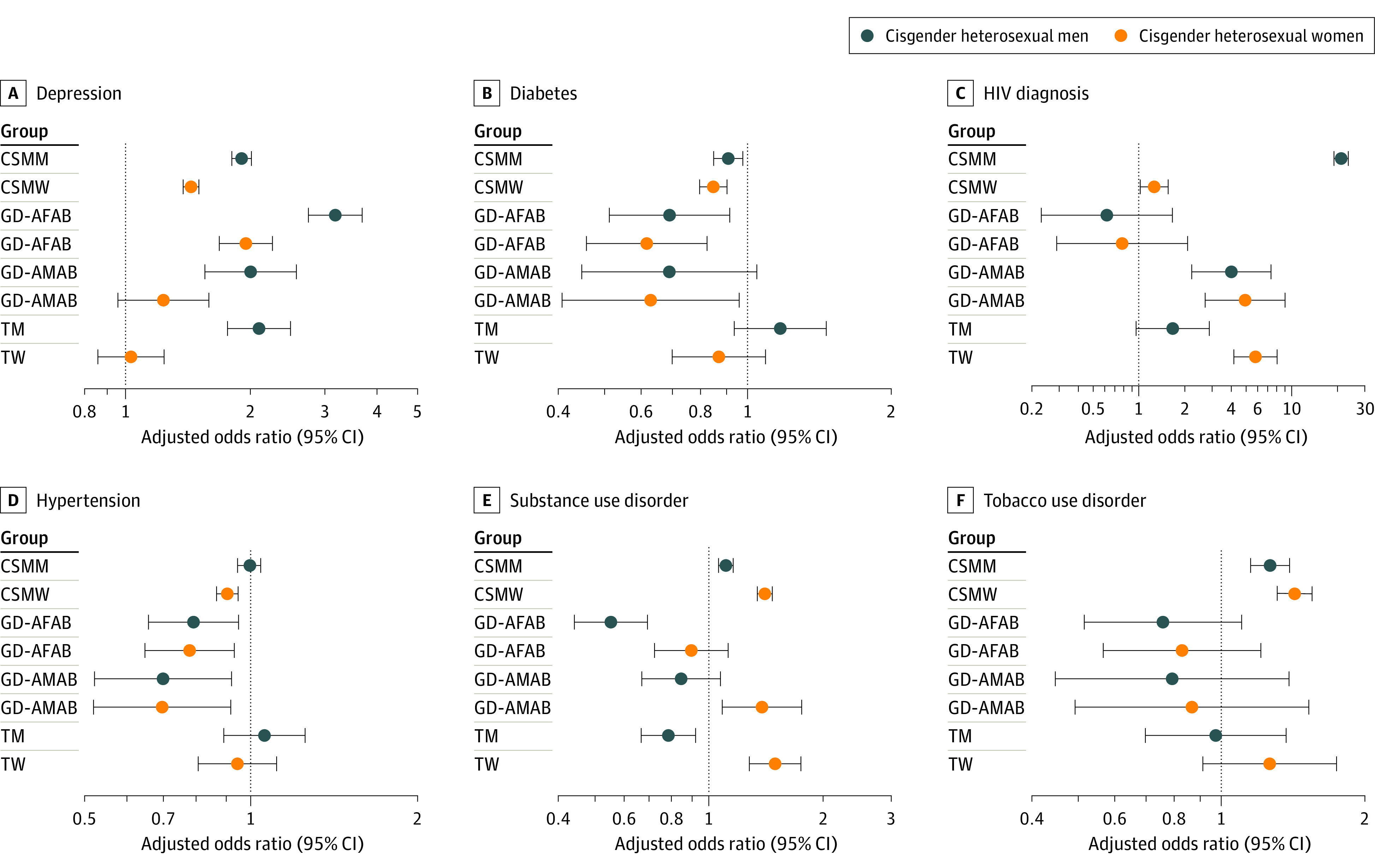
Adjusted Odd Ratios of Depression, Diabetes, HIV Diagnosis, Hypertension, Substance Use Disorder, and Tobacco Use Disorder Among Sexual and Gender Minority Participants in the All of Us Research Program, by Comparison Group Models controlled for current age (continuous), annual income, employment status, enrollment year, and US census division. CSMM indicates cisgender sexual minority men; CSMW, cisgender sexual minority women; GD-AFAB, gender diverse people assigned female at birth of any sexual orientation; GD-AMAB, gender diverse people assigned male at birth of any sexual orientation; TM, transgender men of any sexual orientation; and TW, transgender women of any sexual orientation.

After accounting for age, income, employment status, enrollment year, and US census division, SGM groups more frequently had higher odds of anxiety, depression, HIV diagnosis, and tobacco use disorder but lower odds of cardiovascular disease, kidney disease, diabetes, and hypertension (Figure 2 and Figure 3; eTables 4 to 11 in Supplement 1). Estimated associations for cancer only varied between cisgender sexual minority men (AOR, 1.15; 95% CI, 1.07-1.23) and cisgender sexual minority women (AOR, 0.88; 95% CI, 0.81-0.95) compared with their cisgender heterosexual counterparts (eTable 4 and eTable 5 in Supplement 1). We also found considerable variation by SGM group for asthma, BMI of 25 or higher, and substance use disorder. Gender diverse people AMAB (AOR, 0.39; 95% CI, 0.24-0.63) and transgender women (AOR, 0.51; 95% CI, 0.38-0.69) had lower odds of asthma compared with cisgender heterosexual women although all other SGM groups had higher odds of asthma compared with non-SGM groups (eTables 9 and 11 in Supplement 1). Similarly, gender diverse people AFAB (AOR, 0.35; 95% CI, 0.24-0.52) and transgender men (AOR, 0.65; 95% CI, 0.49-0.87) had lower odds of substance use disorder compared with cisgender heterosexual men (eTables 6 and 10 in Supplement 1). In addition, compared with cisgender heterosexual men, transgender men had higher odds of BMI 25 or higher (AOR, 1.65; 95% CI, 1.38-1.96) (eTable 10 in Supplement 1). Other SGM groups had lower odds compared with non-SGM groups.

Propensity score–weighted models largely yielded consistent findings with regression-adjusted models for 91 of 96 different comparisons (eTables 4 to 11 in Supplement 1). However, where regression models found significant differences, weighted models yielded no significant differences for gender diverse people AFAB compared with cisgender heterosexual men in the odds of cardiovascular disease (AOR, 0.71; 95% CI, 0.48-1.03), diabetes (AOR, 0.86; 95% CI, 0.64-1.14), and hypertension (AOR, 0.91; 95% CI, 0.76-1.10) or between gender diverse people AFAB compared with cisgender heterosexual women in the odds of hypertension (AOR, 0.87; 95% CI, 0.72-1.04) (eTable 6 and eTable 7 in Supplement 1). Compared with cisgender heterosexual women, cisgender sexual minority women had higher odds of BMI 25 or higher (AOR, 1.05; 95% CI, 1.01-1.10) (eTable 5 in Supplement 1).

## Discussion

This cross-sectional study using one of the largest data sets of diverse SGM US adults identified several health inequities that vary by SGM group and health condition. Compared with non-SGM groups, SGM groups showed higher odds of anxiety, depression, HIV diagnosis, and tobacco use disorder but lower odds of cardiovascular disease, kidney disease, diabetes, and hypertension. There were also substantial variations across SGM groups for the odds of asthma, cancer, BMI 25 or higher, and substance use disorder. These findings highlight the need to prioritize SGM populations overall and to address the specific needs of subpopulations within SGM communities. As the All of Us Research Program continues to recruit more participants from historically underrepresented populations, further data disaggregation and subpopulation analyses will enable the study of specific mechanisms affecting health outcomes, particularly for transgender and gender diverse individuals. Overall, these findings demonstrate how the All of Us Research Program’s rich biomedical data can inform research, identify health inequities, and guide policies for health promotion and treatment in SGM populations.

The present study findings support prior work among cisgender sexual minority participants^6,7,8,9,10,11,12,13,14,15^ and research on mental health^18^ and HIV diagnosis^19,52^ among transgender people. However, there are limited data comparing the prevalence of cardiovascular disease, kidney disease, diabetes, and hypertension among transgender and gender diverse participants with cisgender populations. In prior studies using the Behavioral Risk Factor Surveillance System, transgender and gender diverse people showed a higher prevalence of self-reported cardiovascular disease compared with cisgender populations, with no differences reported for the prevalence of diabetes, kidney disease, and hypertension.^17,22,23,28^

However, our findings using the All of Us data may not be directly comparable to prior research. For example, cardiovascular disease and other chronic conditions in the Behavioral Risk Factor Surveillance System are based on self-reported responses.^17^ Unlike in the All of Us program, there were no available data linking self-reported gender identity and sex assigned at birth to EHR data. Additionally, EHR data are currently available only for participants associated with a health care provider organization funded by All of Us, potentially introducing ascertainment bias. Transgender and gender diverse participants who self-selected to enroll in All of Us may differ from their cisgender heterosexual counterparts in factors such as age and access to health care, which can influence cardiovascular disease prevalence and other chronic conditions.^53^ Efforts are being made to improve EHR data transfer and reduce missing data. Future research should consider the impact of differential selection and explore psychosocial (eg, discrimination, psychological distress, and adverse childhood experiences), behavioral (eg, diet, physical activity, alcohol use, and tobacco smoking), and clinical (eg, hypertension, diabetes, HIV, vascular dysfunction, and hormone therapy) factors that may contribute to cardiovascular disease and other chronic conditions among transgender and gender diverse populations.^23^

The associations presented here cannot be assumed to be caused by SGM identity or experiences, as the associations may encompass possible exposure to limited legal protections, discrimination, violence, lack of access to gender-affirming hormone therapy, and lack of robust social networks.^54,55^ These interrelated social and structural factors may be linked to the elevated prevalence of anxiety, asthma, depression, HIV, and substance use observed in this study.^2,3,56,57,58^ Therefore, there is a need to incorporate a theoretical basis in health disparities research that connects the observed differences to mechanisms of structural and interpersonal discrimination^55,57,59^ to identify effective policies and interventions for eliminating health disparities and advancing equity for SGM populations.

The present study is an example of how integrating multiple data sources (eg, health surveys, physical measurements, and EHRs) can provide a novel multidimensional view of SGM people and their health outcomes. This multidimensional view is facilitated by programmatic investments to engage and enroll SGM populations through structured All of Us national community engagement partners^60^ and their activities and key variables that help identify SGM populations (such as employing a 2-step measurement for gender identity and assigned sex at birth).^61,62^ Furthermore, All of Us provides research partners with controlled tier data access to wearable device and genomic data, including whole-genome sequencing. The program also has plans to expand data access to community and international collaborators, issue new surveys, enroll participants in clinical trials, and link All of Us data to other repositories. The cloud-based data analytic platform ensures transparent analytic decisions and enables reproducibility of study findings. These ongoing developments are valuable tools for evaluating interventions to improve the health and well-being of SGM communities.

### Limitations

This study has several limitations. First, it provides an incomplete picture of the health inequities experienced by multiply marginalized groups within SGM communities due to the absence of an intersectional lens.^56,58^ Second, confounding due to place is a potential consideration. Although we were unable to completely account for spatial confounding in our analysis due to the data privacy policies of All of Us, we were able to describe the distribution of the analytic sample by census division and account for this factor in our regression models and propensity score approach with marginal differences in findings between the modeling approaches. Third, the validity of SNOMED diagnostic codes to accurately capture health conditions in All of Us has not yet been tested. Fourth, All of Us relies on convenience sampling and intentionally oversamples underrepresented communities in biomedical research; thus, it is not representative of the US population. Despite these data limitations, large volunteer studies, such as All of Us, provide current estimates based on what is, to our knowledge, the largest sample of SGM people. Moreover, the proportion of SGM adults enrolled in All of Us is comparable to other cross-sectional surveys^1,63^ but has the added strength of the longitudinal collection of data that will be leveraged over time. The ongoing challenge for the program will be to continue to accurately reflect the racial and ethnic, age, and socioeconomic diversity of SGM communities in their cohort.

## Conclusions

This cross-sectional study found that SGM groups showed higher odds of anxiety, depression, HIV diagnosis, and tobacco use disorder, but lower odds of cardiovascular disease, kidney disease, diabetes, and hypertension than non-SGM groups. There were also substantial variations across SGM groups for the odds of asthma, cancer, BMI 25 or higher, and substance use disorder. These results also illustrate the utility of large, diverse, volunteer cohorts with multiple data types in health disparities research, although challenges remain to ensure ongoing engagement with diverse participants and methods development to reduce and address missing data. Future All of Us research examining health inequities should consider conceptual models that connect observable differences to social and structural mechanisms that facilitate these inequities.

## Notes

Supplement 1. **eAppendix.** Acknowledgment List of All of Us Principal Investigators **eTable 1.** Systematized Nomenclature of Medicine—Clinical Terms (SNOMED CT) Codes of Health Conditions **eTable 2.** Survey Responses and Associated Data Generalizations for Sexual Orientation, Gender Identity, and Sex Assigned at Birth by Comparison Group in the All of Us Research Program “Controlled Tier” Data Set **eFigure 1.** Percentage of Missing Body Mass Index and Electronic Health Record Data by Sexual Orientation and Gender Identity Groups **eTable 3.** Participant Characteristics of the Overall All of Us Research Program Cohort, Participants With BMI, and Participants With EHR Data **eFigure 2.** Propensity Score Distribution Between Sexual and Gender Minority Groups (SGM) Compared To Their Non-SGM Counterparts **eFigure 3.** Absolute Standardized Mean Differences Between A) Cisgender Sexual Minority Men Compared to Cisgender Heterosexual Men, B) Cisgender Sexual Minority Women Compared to Cisgender Heterosexual Women, C) Gender Diverse People Assigned Female at Birth (of Any Sexual Orientation) Compared to Cisgender Heterosexual Men, and D) Gender Diverse People Assigned Female at Birth (of Any Sexual Orientation) Compared to Cisgender Heterosexual Women **eFigure 4.** Absolute Standardized Mean Differences Between E) Gender Diverse People Assigned Male at Birth (of Any Sexual Orientation) Compared to Cisgender Heterosexual Men, F) Gender Diverse People Assigned Male at Birth (of Any Sexual Orientation) Compared to Cisgender Heterosexual Women, G) Transgender Men (of Any Sexual Orientation) Compared to Cisgender Heterosexual Men, and H) Transgender Women (of Any Sexual Orientation) Compared to Cisgender Heterosexual Women **eTable 4.** Estimated Odds Ratios of 12 Health Conditions Between Cisgender Sexual Minority Men and Cisgender Heterosexual Men in the All of Us Research Program **eTable 5.** Estimated Odds Ratios of 12 Health Conditions Between Cisgender Sexual Minority Women and Cisgender Heterosexual Women in the All of Us Research Program **eTable 6.** Estimated Odds Ratios of 12 Health Conditions Between Gender Diverse People Assigned Female at Birth of Any Sexual Orientation and Cisgender Heterosexual Men in the All of Us Research Program **eTable 7.** Estimated Odds Ratios of 12 Health Conditions Between Gender Diverse People Assigned Female at Birth of Any Sexual Orientation and Cisgender Heterosexual Women in the All of Us Research Program **eTable 8.** Estimated Odds Ratios of 12 Health Conditions Between Gender Diverse People Assigned Male at Birth of Any Sexual Orientation and Cisgender Heterosexual Men in the All of Us Research Program **eTable 9.** Estimated Odds Ratios of 12 Health Conditions Between Gender Diverse People Assigned Male at Birth of Any Sexual Orientation and Cisgender Heterosexual Women in the All of Us Research Program **eTable 10.** Estimated Odds Ratios of 12 Health Conditions Between Transgender Men of Any Sexual Orientation and Cisgender Heterosexual Men in the All of Us Research Program **eTable 11.** Estimated Odds Ratios of 12 Health Conditions Between Transgender Women of Any Sexual Orientation and Cisgender Heterosexual Women in the All of Us Research Program

Supplement 2.
Data Sharing Statement
