# Supplementary material for: Prevalence of 12 Common Health Conditions in Sexual and Gender Minority Participants in the All of Us Research Program
Source: JAMA Netw Open. 2023 Jul 31;6(7):e2324969. doi: 10.1001/jamanetworkopen.2023.24969 (PMC10391317; doi:10.1001/jamanetworkopen.2023.24969)
Supplement: Supplement 1. — eAppendix. Acknowledgment List of All of Us Principal Investigators eTable 1. Systematized Nomenclature of Medicine—Clinical Terms (SNOMED CT) Codes of Health Conditions eTable 2. Survey Responses and Associated Data Generalizations for Sexual Orientation, Gender Identity, and Sex Assigned at Birth by Comparison Group in the All of Us Research Program “Controlled Tier” Data Set eFigure 1. Percentage of Missing Body Mass Index and Electronic Health Record Data by Sexual Orientation and Gender Identity Groups eTable 3. Participant Characteristics of the Overall All of Us Research Program Cohort, Participants With BMI, and Participants With EHR Data eFigure 2. Propensity Score Distribution Between Sexual and Gender Minority Groups (SGM) Compared To Their Non-SGM Counterparts eFigure 3. Absolute Standardized Mean Differences Between A) Cisgender Sexual Minority Men Compared to Cisgender Heterosexual Men, B) Cisgender Sexual Minority Women Compared to Cisgender Heterosexual Women, C) Gender Diverse People Assigned Female at Birth (of Any Sexual Orientation) Compared to Cisgender Heterosexual Men, and D) Gender Diverse People Assigned Female at Birth (of Any Sexual Orientation) Compared to Cisgender Heterosexual Women eFigure 4. Absolute Standardized Mean Differences Between E) Gender Diverse People Assigned Male at Birth (of Any Sexual Orientation) Compared to Cisgender Heterosexual Men, F) Gender Diverse People Assigned Male at Birth (of Any Sexual Orientation) Compared to Cisgender Heterosexual Women, G) Transgender Men (of Any Sexual Orientation) Compared to Cisgender Heterosexual Men, and H) Transgender Women (of Any Sexual Orientation) Compared to Cisgender Heterosexual Women eTable 4. Estimated Odds Ratios of 12 Health Conditions Between Cisgender Sexual Minority Men and Cisgender Heterosexual Men in the All of Us Research Program eTable 5. Estimated Odds Ratios of 12 Health Conditions Between Cisgender Sexual Minority Women and Cisgender Heterosexual Women in the [file jamanetwopen-e2324969-s001.pdf]

Supplementary Online Content

Tran NK, Lunn MR, Schulkey CE, et al. Prevalence of 12 common health conditions in sexual and gender minority participants in the All of Us Research Program. *JAMA Netw Open*. 2023;6(7):e2324969. doi:10.1001/jamanetworkopen.2023.24969

eAppendix. Acknowledgement List of All of Us Principal Investigators

eTable 1. Systematized Nomenclature of Medicine–Clinical Terms (SNOMED CT) Codes of Health Conditions

eTable 2. Survey Responses and Associated Data Generalizations for Sexual Orientation, Gender Identity, and Sex Assigned at Birth by Comparison Group in the All of Us Research Program “Controlled Tier” Data Set

eFigure 1. Percentage of Missing Body Mass Index and Electronic Health Record Data by Sexual Orientation and Gender Identity Groups

eTable 3. Participant Characteristics of the Overall All of Us Research Program Cohort, Participants With BMI, and Participants With EHR Data

eFigure 2. Propensity Score Distribution Between Sexual and Gender Minority Groups (SGM) Compared To Their Non-SGM Counterparts

eFigure 3. Absolute Standardized Mean Differences Between A) Cisgender Sexual Minority Men Compared to Cisgender Heterosexual Men, B) Cisgender Sexual Minority Women Compared to Cisgender Heterosexual Women, C) Gender Diverse People Assigned Female at Birth (of Any Sexual Orientation) Compared to Cisgender Heterosexual Men, and D) Gender Diverse People Assigned Female at Birth (of Any Sexual Orientation) Compared to Cisgender Heterosexual Women

eFigure 4. Absolute Standardized Mean Differences Between E) Gender Diverse People Assigned Male at Birth (of Any Sexual Orientation) Compared to Cisgender Heterosexual Men, F) Gender Diverse People Assigned Male at Birth (of Any Sexual Orientation) Compared to Cisgender Heterosexual Women, G) Transgender Men (of Any Sexual Orientation) Compared to Cisgender Heterosexual Men, and H) Transgender Women (of Any Sexual Orientation) Compared to Cisgender Heterosexual Women

eTable 4. Estimated Odds Ratios of 12 Health Conditions Between Cisgender Sexual Minority Men and Cisgender Heterosexual Men in the All of Us Research Program

eTable 5. Estimated Odds Ratios of 12 Health Conditions Between Cisgender Sexual Minority Women and Cisgender Heterosexual Women in the All of Us Research Program

eTable 6. Estimated Odds Ratios of 12 Health Conditions Between Gender Diverse People Assigned Female at Birth of Any Sexual Orientation and Cisgender Heterosexual Men in the All of Us Research Program

eTable 7. Estimated Odds Ratios of 12 Health Conditions Between Gender Diverse People Assigned Female at Birth of Any Sexual Orientation and Cisgender Heterosexual Women in the All of Us Research Program

eTable 8. Estimated Odds Ratios of 12 Health Conditions Between Gender Diverse People Assigned Male at Birth of Any Sexual Orientation and Cisgender Heterosexual Men in the All of Us Research Program

© 2023 Tran NK et al. *JAMA Network Open.*

eTable 9. Estimated Odds Ratios of 12 Health Conditions Between Gender Diverse People Assigned Male at Birth of Any Sexual Orientation and Cisgender Heterosexual Women in the All of Us Research Program

eTable 10. Estimated Odds Ratios of 12 Health Conditions Between Transgender Men of Any Sexual Orientation and Cisgender Heterosexual Men in the All of Us Research Program

eTable 11. Estimated Odds Ratios of 12 Health Conditions Between Transgender Women of Any Sexual Orientation and Cisgender Heterosexual Women in the All of Us Research Program

This supplementary material has been provided by the authors to give readers additional information about their work.

eAPPENDIX. Acknowledgement List Of *All Of Us* Principal Investigators

Past and Present *All of Us* Research Program Principal Investigators

Brian Ahmedani, PhD, MSW[1]; Christine D Cole Johnson, PhD, MPH[1]; Habib Ahsan, MD, MMedSc[2]; Donna Antoine-LaVigne, PhD, MPH, MSEd*[3]; Glendora Singleton*[3]; Pamelia Watson-McGee[3]; Arnita Ford Norwood, PhD, MPH, RDN[3]; Hoda Anton-Culver, PhD[4]; Eric Topol, MD[5]; Katie Baca-Motes, MBA[5]; Julia Moore-Vogel, PhD, MBA[5]; Steven Steinhubl, MD*[5]; Praduman Jain, MSEE[6]; Mark Begale[6]; Neeta Jain[6]; David Klein, MBA[6]; Scott Sutherland[6]; James Wade, MD*[6]; Bruce Korf, MD, PhD[7]; Mona Fouad, MD, PhD[7]; Beth Lewis[7]; David B Goldstein, PhD[8]; Louise Bier, MS[8]; Ali G Gharavi, MD[8]; George Hripcsak, MD, MS[8]; Eric Boerwinkle, PhD, MS, MA[9]; Murray H Brilliant, PhD*[10]; Narayana Murali[10]; Scott Joseph Hebbring[10]; Elizabeth Burnside[11]; Dorothy Farrar-Edwards, PhD[11]; Yashoda Sharma, PhD[12]; Amy Taylor[12]; Chinea, MD, MPH*[13]; Liliana Lombardi Desa[13]; Nancy Jenks, MS, CFNP, FAANP[13]; Steve Thibodeau[14]; Mine Cicek, PhD[14]; Eric Schlueter, MD[15]; Beverly Wilson Holmes, MSW[15]; Martha Daviglus, MD, PhD[16]; Robert Winn, MD*[16]; Paul Harris, PhD[+17]; Consuelo Wilkins, MD, MSCI[17]; Dan Roden, MD, CM[17]; Joshua Denny, MD, MS*[17]; Kim Doheny[18]; Debbie Nickerson, PhD[19]; Evan Eichler[19]; Gail Jarvik, MD, PhD[19]; Gretchen Funk[20]; Sallie Hussey[20]; Anthony Philippakis, MD, PhD[21]; Heidi Rehm, PhD, MMSc, FACMG[21]; Stacey Gabriel, PhD[21]; Richard Gibbs[22]; Edgar M Gil Rico, MBA, MSc[23]; David Glazer[24]; Jessica Burke, MBA[25]; Philip Greenland, MD[26]; Elizabeth Shenkman, PhD[27]; William R Hogan, MD, MS[27]; Priscilla Igho-Pemu, MD, MSCR, FACP[28]; W Karlson, MD[29]; Jordan Smoller, MD, ScD[29]; Shawn N Murphy, MD, PhD[29]; Margaret Elizabeth Ross, MD, PhD[30]; Rainu Kaushal, MD, MPH[30]; Eboni Winford, PhD[31]; Febe Wallace, MD[31]; Parinda Khatri, PhD[31]; Vik Kheterpal[32]; Monica Kraft[33]; Francisco A Moreno, MD[33]; Irving Kron*[33]; Rachele Peterson, MS*[33]; Patricia Watkins Lattimore*[34]; Cheryl Thomas[34]; Mitchell Lunn, MD, MAS, FASN[35]; Juno Obedin-Maliver, MD, MPH, MAS[35]; Oscar Marroquin, MD[36]; Shyam Visweswaran, MD, PhD[36]; Steven Reis, MD[36]; Patrick McGovern[37]; Fatima Munoz, MD, MPH[38]; Gregory Talavera, MD, MPH[38]; George T O'Connor, MD, MS[39]; Christopher O'Donnell, MD, MPH*[40]; Lucila Ohno-Machado, MD, PhD[41]; Greg Orr*[42]; Fornessa Randal, MCRP[43]; Andreas A Theodorou, MD[44]; Eric Reiman, MD[44]; Mercedita Roxas-Murray[45]; Louisa Stark[46]; Ronnie Tepp, MPP[47]; Alicia Zhou, PhD[48]; Scott Topper, PhD, FACMG[48]; Rhonda Trousdale, MD[49]; Phil Tsao, PhD[50]; Scott T Weiss, MD, MS[51]; David Wellis, PhD[52]; Jeffrey Whittle, MD, MPH[53]; Amanda Wilson, MS[54]; Stephan Zuchner, MD, PhD[55]; Olveen Carrasquillo, MD, PhD[55]; Margaret Pericak-Vance[55]; Michael E Zwick, PhD[56]; Megan Lewis[57]; Jen Uhrig[57]; May Okihiro[58]

Note: This is the list of individuals who were Principal Investigators or equivalent with the *All of Us* Research Program during the period that this paper was in development (October 25, 2022 – January 6, 2023).

Legend
*Past Principal Investigator
+Principal Investigator/Lead Author for the *All of Us* Research Program protocol (paul.a.harris@vumc.org)

Affiliations
1. Henry Ford Health System
2. University of Chicago Medical Center
3. Jackson-Hinds Comprehensive Health Center
4. University of California, Irvine
5. Scripps Research Translational Institute

© 2023 Tran NK et al. *JAMA Network Open.*

6. Vibrent Health
7. University of Alabama at Birmingham
8. Columbia University
9. University of Texas Health Science Center at Houston
10. Marshfield Clinic Research Institute
11. University of Wisconsin at Madison
12. Community Health Center, Inc.
13. Sun River Health
14. Mayo Clinic and Foundation, Rochester
15. Cooperative Health
16. University of Illinois at Chicago
17. Vanderbilt University Medical Center
18. Johns Hopkins University School of Medicine
19. University of Washington
20. FiftyForward
21. Broad Institute
22. Baylor University
23. National Alliance for Hispanic Health
24. Verily Life Sciences
25. MITRE Corporation
26. Northwestern University
27. University of Florida
28. Morehouse School of Medicine, Atlanta
29. Partners Health Care
30. Cornell University, Weill Medical College
31. Cherokee Health Systems
32. CareEvolution, Inc.
33. University of Arizona, Tucson
34. Delta Research and Educational Foundation
35. Stanford University
36. University of Pittsburgh
37. Wondros
38. San Ysidro Health Center
39. Boston Medical Center
40. VA *All of Us* Coordinating Center, Boston
41. University of California, San Diego
42. Walgreen Co.
43. Asian Health Coalition
44. Banner Health
45. Montage Marketing Group
46. University of Utah
47. HCM Strategists
48. Color Genomics, Inc.
49. NYC Health + Hospitals
50. VA AoU Coordinating Center - Palo Alto
51. Brigham and Women's Hospital
52. San Diego Blood Bank
53. Medical College of Wisconsin
54. National Library of Medicine (NLM)
55. University of Miami School of Medicine
56. Emory University

© 2023 Tran NK et al. *JAMA Network Open.*

57. Research Triangle Institute
58. Waianae Coast CHC

© 2023 Tran NK et al. *JAMA Network Open.*

SUPPLEMENTARY TABLES & FIGURES

eTable 1. Systematized Nomenclature of Medicine – Clinical Terms (SNOMED CT) Codes of Health Conditions.

Health Condition	SNOMED CT Name	SNOMED CT Code
Anxiety	Anxiety	48694002
Asthma	Asthma	195967001
Cancer	Malignant neoplastic disease	363346000
Cardiovascular disease	Coronary arteriosclerosis	53741008
	Myocardial infarction	22298006
	Heart failure	84114007
Chronic kidney disease	Chronic kidney disease	709044004
Depression	Depressive Disorder	35489007
Diabetes mellitus	Diabetes mellitus	73211009
HIV diagnosis	Human immunodeficiency virus infection	86406008
Hypertension	Hypertensive disorder	38341003
Substance use disorder	Substance abuse	66214007
Tobacco use disorder	Tobacco dependence syndrome	89765005

© 2023 Tran NK et al. *JAMA Network Open.*

eTable 2. Survey Responses and Associated Data Generalizations for Sexual Orientation, Gender Identity, and Sex Assigned at Birth by Comparison Group in the *All of Us* Research Program "Controlled Tier" Dataset

Comparison Group	Gender Identity[a] Participant Survey Response	Sex Assigned at Birth[b] Survey Response	Sexual Orientation[c] Participant Survey Response
Cisgender heterosexual women	"Woman" only	Female	• "Straight; that is not gay or lesbian, etc." only
Cisgender heterosexual men	"Man" only	Male	• "Straight; that is not gay or lesbian, etc." only
Cisgender sexual minority women	"Woman" only	Female	Anyone who answered the sexual orientation item (*i.e.*, not skipped, not missing) and answered anything **<u>other than</u>** the following answer choices alone or in any combination with each other:<br>• "Straight; that is not gay or lesbian, etc."<br>• "Have not figured out or are in the process of figuring out your sexuality"<br>• "Do not think of yourself as having sexuality"<br>• "Do not use labels to identify yourself"<br>• "Don't know the answer"<br>"Prefer not to answer"
Cisgender sexual minority men	"Man" only	Male	Anyone who answered the sexual orientation item (*i.e.*, not skipped, not missing) and answered anything **<u>other than</u>** the following answer choices alone or in any combination with each other:<br>• "Straight; that is not gay or lesbian, etc."<br>• "Have not figured out or are in the process of figuring out your sexuality"<br>• "Do not think of yourself as having sexuality"<br>• "Do not use labels to identify yourself"

© 2023 Tran NK et al. *JAMA Network Open.*

Comparison Group	Gender Identity[a] Participant Survey Response	Sex Assigned at Birth[b] Survey Response	Sexual Orientation[c] Participant Survey Response
			• "Don't know the answer"<br>"Prefer not to answer"
Gender-diverse people assigned male sex at birth (of any sexual orientation)	Anyone who answered the gender identity item (*i.e.*, not skipped, not missing) and did **not** select "prefer not to answer" only and does **not** fit into any of the other groups	Male	Any
Gender-diverse people assigned female sex at birth (of any sexual orientation)	Anyone who answered the gender identity item (*i.e.*, not skipped, not missing) and did **not** select "prefer not to answer" only and does **not** fit into any of the other groups	Female	Any
Transgender men (of any sexual orientation)	• "Man" only<br>• "Transgender" only<br>• "Trans man/Transgender Man/FTM" only<br>Any combination of the 3 selections above	Female	Any
Transgender women (of any sexual orientation)	• "Woman" only<br>• "Transgender" only<br>• "Trans woman/Transgender Woman/MTF" only<br>Any combination of the 3 selections above	Male	Any

[a] Participants could select any (or multiple) responses including man, woman, non-binary, transgender, none of these describe me and I'd like to consider additional options, and/or prefer not to answer. Participants who endorsed non-binary, transgender, or none of these describe me and I'd like to consider additional options were shown the following response options: trans man/transgender man/FTM, trans woman/transgender women/MTF, genderqueer, genderfluid, gender variant, Two-spirit, questioning or unsure of gender identity, and/or none of these describe me, and I want to specify.

[b] For sex assigned at birth, participants were excluded if they answered "intersex," "prefer not to answer," or skipped the question.

[c] Participants could select any (or multiple) responses including gay, lesbian, straight; that is, not gay or lesbian, etc, bisexual, and/or none of these describe me and I'd like to consider additional options. Participants who endorsed none of these describe me and I'd like to consider additional options were shown the following responses: queer; polysexual, omnisexual, sapiosexual or pansexual; asexual; Two-spirit; have not figure out or in the process of figuring out your sexuality; mostly straight, but sometimes attracted to people of your own sex; do not think of yourself as having sexuality; do not use labels to identity yourself; don't know the answer; no I mean something else, please specify; and/or prefer not to answer.

© 2023 Tran NK et al. *JAMA Network Open.*

eFigure 1. Percentage of Missing Body Mass Index and Electronic Health Record Data by Sexual Orientation and Gender Identity Groups

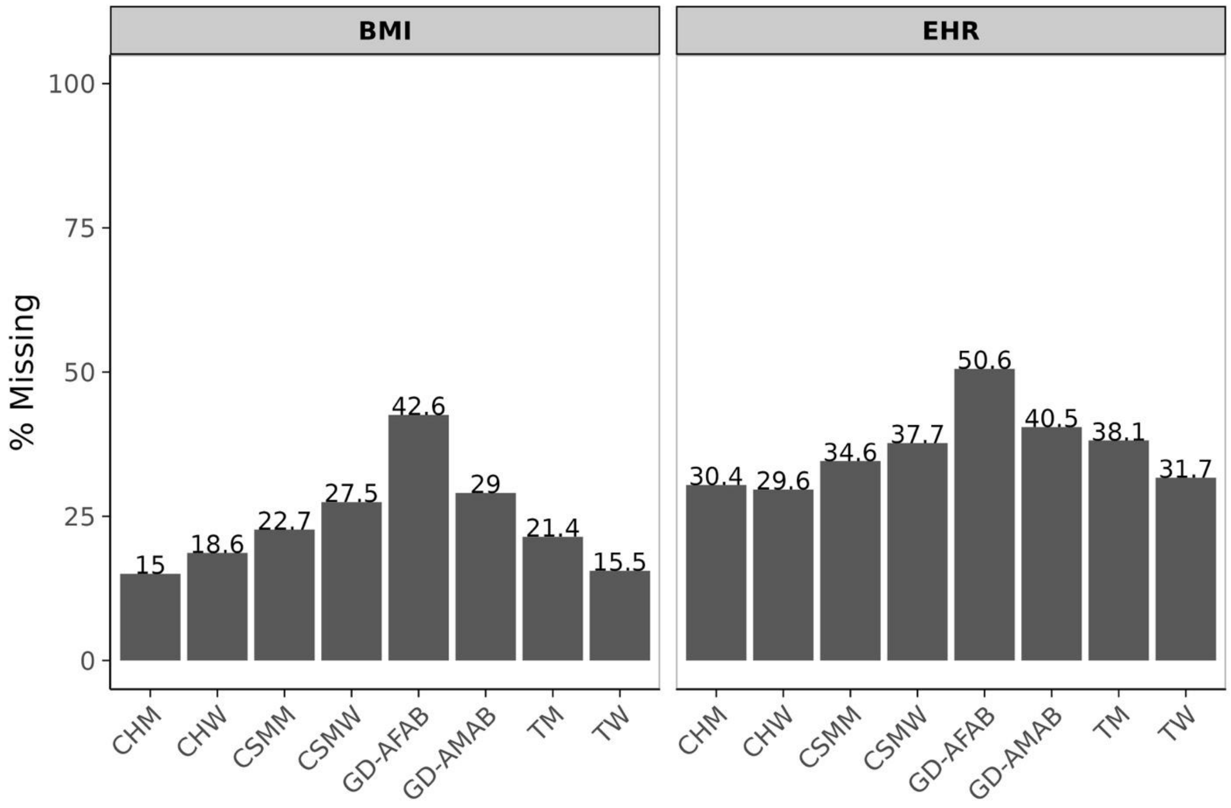

BMI: Body mass index; EHR: Electronic health record; CHM: Cisgender heterosexual men; CHW: Cisgender heterosexual women; CSMM: Cisgender sexual minority men; CSWM: Cisgender sexual minority women; GE-AFAB: Gender diverse people assigned female at birth of any sexual orientation; GE-AMAB: Gender diverse people assigned male at birth of any sexual orientation; TM: Transgender men of any sexual orientation; TW: Transgender women of any sexual orientation.

© 2023 Tran NK et al. *JAMA Network Open.*

eTable 3. Participant Characteristics of the Overall *All of Us* Research Program Cohort, Participants With BMI, and Participants With EHR data

	No. (%)		
	Overall cohort	Participants with BMI	Participants with EHR
**Categorical variables**	**n = 346,868**	**n = 278,128**	**n = 240,841**
Gender Identity and Sexual Orientation Groups			
Cisgender heterosexual men	120,568 (34.8)	100,398 (36.1)	83,890 (34.8)
Cisgender heterosexual women	195,537 (56.4)	155,364 (55.9)	137,593 (57.1)
Cisgender sexual minority men	10,980 (3.2)	8,350 (3.0)	7,183 (3.0)
Cisgender sexual minority women	16,096 (4.6)	11,462 (4.1)	10,028 (4.2)
Gender diverse people AFAB of any sexual orientation	1,428 (0.4)	803 (0.3)	706 (0.3)
Gender diverse people AMAB of any sexual orientation	482 (0.1)	332 (0.1)	287 (0.1)
Transgender men of any sexual orientation	928 (0.3)	716 (0.3)	574 (0.2)
Transgender women of any sexual orientation	849 (0.3)	703 (0.3)	580 (0.2)
Ethnoracial Identity[a]			
African American or Black	70,755 (20.4)	63,560 (22.8)	51,793 (21.5)
Asian	14,237 (4.1)	10,758 (3.9)	8,364 (3.5)
Hispanic or Latinx	62,357 (18.0)	54,740 (19.7)	45,507 (18.9)
Middle Eastern or North African	3,647 (1.1)	2,820 (1.0)	2,460 (1.0)
Native Hawaiian or other Pacific Islander	880 (0.3)	729 (0.3)	593 (0.2)
White	201,128 (58.0)	149,996 (53.9)	135,847 (56.4)
Sexual Orientation[a]			
Asexual	547 (0.2)	352 (0.1)	299 (0.1)
Bisexual	13,242 (3.8)	9,449 (3.4)	8,187 (3.4)
Gay	8,393 (2.4)	6,304 (2.3)	5,479 (2.3)
Lesbian	4,.650 (1.3)	3,386 (1.2)	2,997 (1.2)
Mostly straight	699 (0.2)	469 (0.2)	405 (0.2)
Queer	764 (0.2)	434 (0.2)	355 (0.1)
Polysexual, omnisexual, sapiosexual or pansexual	733 (0.2)	430 (0.2)	396 (0.2)
Straight	318,037 (91.7)	257,175 (92.5)	222,681 (92.5)
Two-spirit	64 (<0.01)	58 (<0.01)	47 (<0.01)
Gender Identity[a]			
Genderfluid, genderqueer, gender variant, unsure, specific gender, or Two-spirit	259 (0.1)	180 (0.1)	158 (0.1)
Man	132,031 (38.1)	109,169 (39.3)	91,409 (38.0)
Non-binary	1616 (0.5)	931 (0.3)	814 (0.3)
Transgender	1079 (0.3)	685 (0.2)	563 (0.2)
Woman	212018 (61.1)	167,156 (60.1)	147,901 (61.4)
Annual Household Income			
Less than $25,000	89,568 (25.8)	77,671 (27.9)	64,757 (26.9)
$25,000-$49,999	52,893 (15.2)	41,296 (14.8)	36,363 (15.1)
$50,000-$99,999	65,892 (19.0)	49,004 (17.6)	43,863 (18.2)
$100,000-$149,999	35,262 (10.2)	25,676 (9.2)	22,942 (9.5)
≥$150,000	38,991 (11.2)	28,058 (10.1)	25,280 (10.5)
Prefer to not answer or skipped	64,262 (18.5)	56,426 (20.3)	47,636 (19.8)
Education levels			
HS graduate or less	97,102 (28.0)	86,374 (31.1)	71,846 (29.8)
Some college	89,039 (25.7)	71,132 (25.6)	62,563 (26.0)
4-year College graduate	79,155 (22.8)	60,363 (21.7)	53,189 (22.1)
Advanced degree	75,622 (21.8)	55,033 (19.8)	49,065 (20.4)
Prefer not to answer or skipped	5,950 (1.7)	5,226 (1.9)	4,178 (1.7)
Employed for wages			
No	190,674 (55.0)	158,885 (57.1)	137,691 (57.2)
Yes	147,323 (42.5)	111,456 (40.1)	96,947 (40.3)
Prefer not to answer or skipped	8,871 (2.6)	7,787 (2.8)	6,203 (2.6)
Owned a home			
No	174,020 (50.2)	144,817 (52.1)	122,381 (50.8)
Yes	159,853 (46.1)	121,964 (43.9)	109,240 (45.4)
Prefer not to answer or skipped	12,995 (3.7)	11,347 (4.1)	9,220 (3.8)

© 2023 Tran NK et al. *JAMA Network Open.*

Insured			
No	22,905 (6.6)	20,054 (7.2)	15,058 (6.3)
Yes	316,190 (91.2)	251,364 (90.4)	220,600 (91.6)
Prefer not to answer or skipped	7773 (2.2)	6,710 (2.4)	5,289 (2.2)
PROMIS GPH: Poor/Fair[b]			
No	225,962 (65.1)	182,290 (65.5)	156,737 (65.1)
Yes	69,112 (20.0)	57,350 (20.6)	52,548 (21.8)
Prefer not to answer or skipped	51,794 (14.9)	38,488 (13.8)	31,556 (13.1)
PROMIS GMH: Poor/Fair[b]			
No	253,641 (73.1)	207,750 (74.7)	179,539 (74.5)
Yes	50,033 (14.4)	39,939 (14.4)	35,598 (14.8)
Prefer not to answer or skipped	43,192 (12.5)	30,439 (10.9)	25,704 (10.7)
AUDIT-C Score ≥3[c]			
No	209,447 (60.4)	174,100 (62.6)	152,210 (63.2)
Yes	123,075 (35.5)	99,323 (35.7)	85,410 (35.5)
Prefer not to answer or skipped	14,346 (4.1)	4,705 (1.7)	3,221 (1.3)
AUDIT-C Score ≥4[c]			
No	248,893 (71.8)	205,987 (74.1)	179,821 (74.7)
Yes	83,629 (24.1)	67,436 (24.2)	57,799 (24.0)
Prefer not to answer or skipped	14,346 (4.1)	4,705 (1.7)	3,221 (1.3)
Substance Use, 3 months[d]			
No	286,759 (82.7)	234,688 (84.4)	205,128 (85.2)
Yes	30,313 (8.7)	25,623 (9.2)	21,317 (8.9)
Prefer not to answer or skipped	29,796 (8.6)	17,817 (6.4)	14,396 (6.0)
Current Smoker			
No	271,442 (78.3)	219,721 (79.0)	193,295 (80.3)
Yes	56.260 (16.2)	49,711 (17.9)	40,860 (17.0)
Prefer not to answer or skipped	19,166 (5.5)	8,696 (3.1)	6,686 (2.8)
Enrollment year			
2017-2018	104,335 (30.1)	85,361 (30.7)	75,046 (31.2)
2019	154,352 (44.5)	134,318 (48.3)	112,345 (46.6)
2020	46,983 (13.5)	32,944 (11.8)	28,710 (11.9)
2021-2022	41,198 (11.9)	25,505 (9.2)	24,740 (10.3)
Divisions			
East North Central	70,169 (20.2)	56,228 (20.2)	49,744 (20.7)
East South Central	26,974 (7.8)	22,709 (8.2)	17,173 (7.1)
Middle Atlantic	63,192 (18.2)	53,449 (19.2)	49,576 (20.6)
Mountain	46,596 (13.4)	37,963 (13.6)	34,775 (14.4)
New England	33,939 (9.8)	27,638 (9.9)	27,013 (11.2)
Pacific	49,838 (14.4)	40,304 (14.5)	30,446 (12.6)
South Atlantic	332,782 (9.5)	23,511 (8.5)	20,893 (8.7)
West North Central	7,167 (2.1)	3,981 (1.4)	2,908 (1.2)
West South Central	16,007 (4.6)	12,220 (4.4)	8,208 (3.4)
US Territory or missing	204 (0.01)	125 (<0.01)	105 (<0.01)
**Continuous variables**			
Age, mean (SD)	53.9 (17.1)	54.4 (17.0)	55.0 (17.0)
Age, median (IQR)	55 (39, 68)	56 (40, 68)	57 (41, 68)

AFAB: Assigned female at birth; AMAB: Assigned male at birth; SD: standard deviation; IQR: Interquartile range; PROMIS: Patient-Reported Outcomes Measurement Information System; GPH: Global physical health; GMH: Global mental health; AUDIT-C: Alcohol Use Disorders Identification Test-Concise

[a] Categories are not mutually exclusive; they do not sum to the column total because participants may self-identify in multiple groups.

[b] Scores of less than 40 for GPH and less than 42 for GMH were considered Poor or Fair.

[c] Scores of 3 or greater were used as the cutoff for women, scores of 4 or greater were used as the cutoff for men, and both cutoffs were used for gender diverse people. Cutoffs indicated screening positive for past-year hazardous drinking.

© 2023 Tran NK et al. *JAMA Network Open.*

[d] Included cannabis, cocaine, prescription and non-prescription stimulants, inhalants, sedatives, hallucinogens, or prescription and nonprescription opioids.

© 2023 Tran NK et al. *JAMA Network Open.*

eFigure 2. Propensity Score Distribution Between Sexual and Gender Minority Groups (SGM) Compared to Their Non-SGM Counterparts. AFAB: Assigned Female At Birth. AMAB: assigned male at birth.

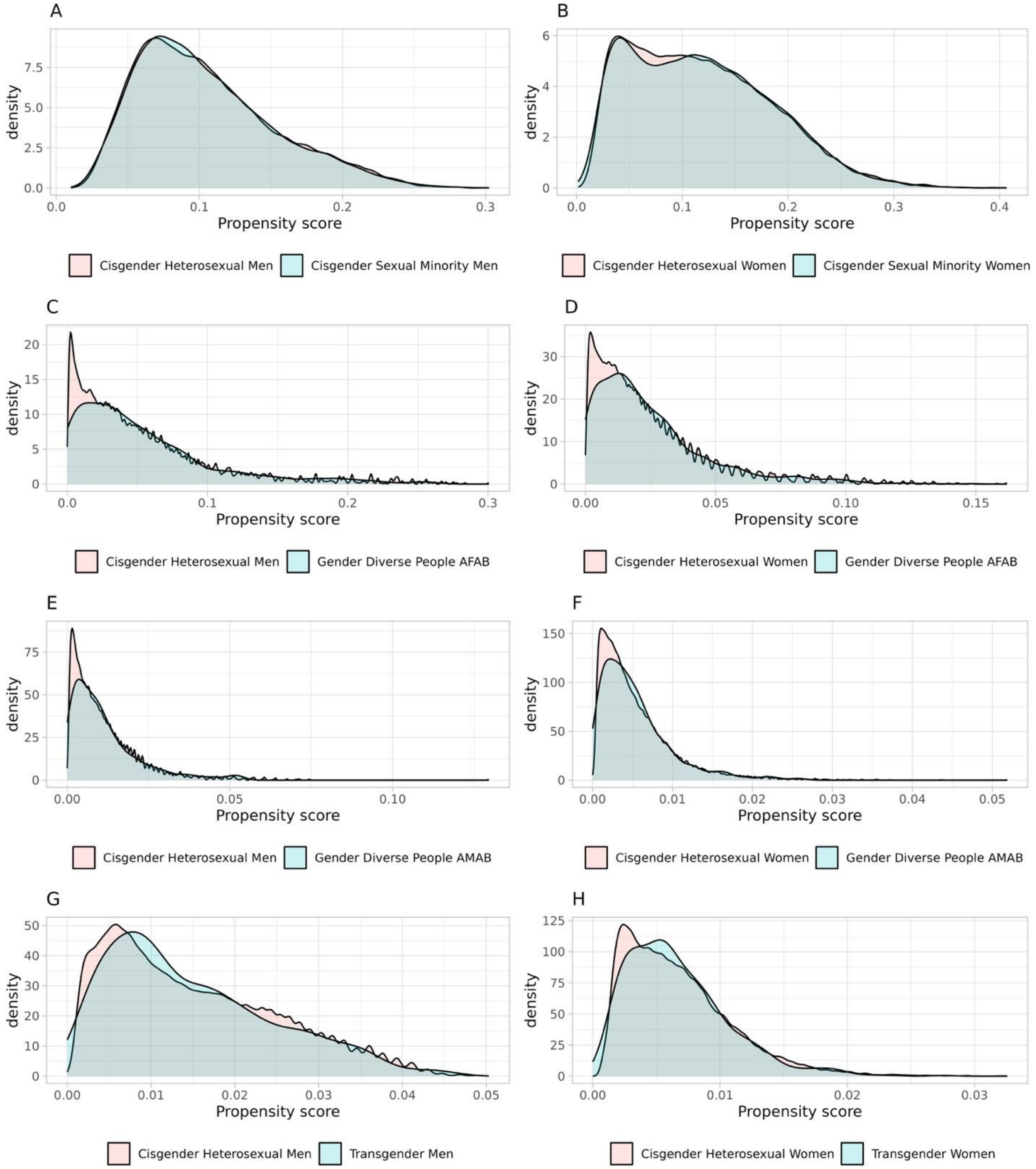

© 2023 Tran NK et al. *JAMA Network Open.*

eFigure 3. Absolute standardized Mean Differences Between A) Cisgender Sexual Minority Men Compared to Cisgender Heterosexual Men, B) Cisgender Sexual Minority Women Compared to Cisgender Heterosexual Women, C) Gender Diverse People Assigned Female at Birth (of Any Sexual Orientation) Compared to Cisgender Heterosexual Men, and D) Gender Diverse People Assigned Female at Birth (of Any Sexual Orientation) Compared to Cisgender Heterosexual Women

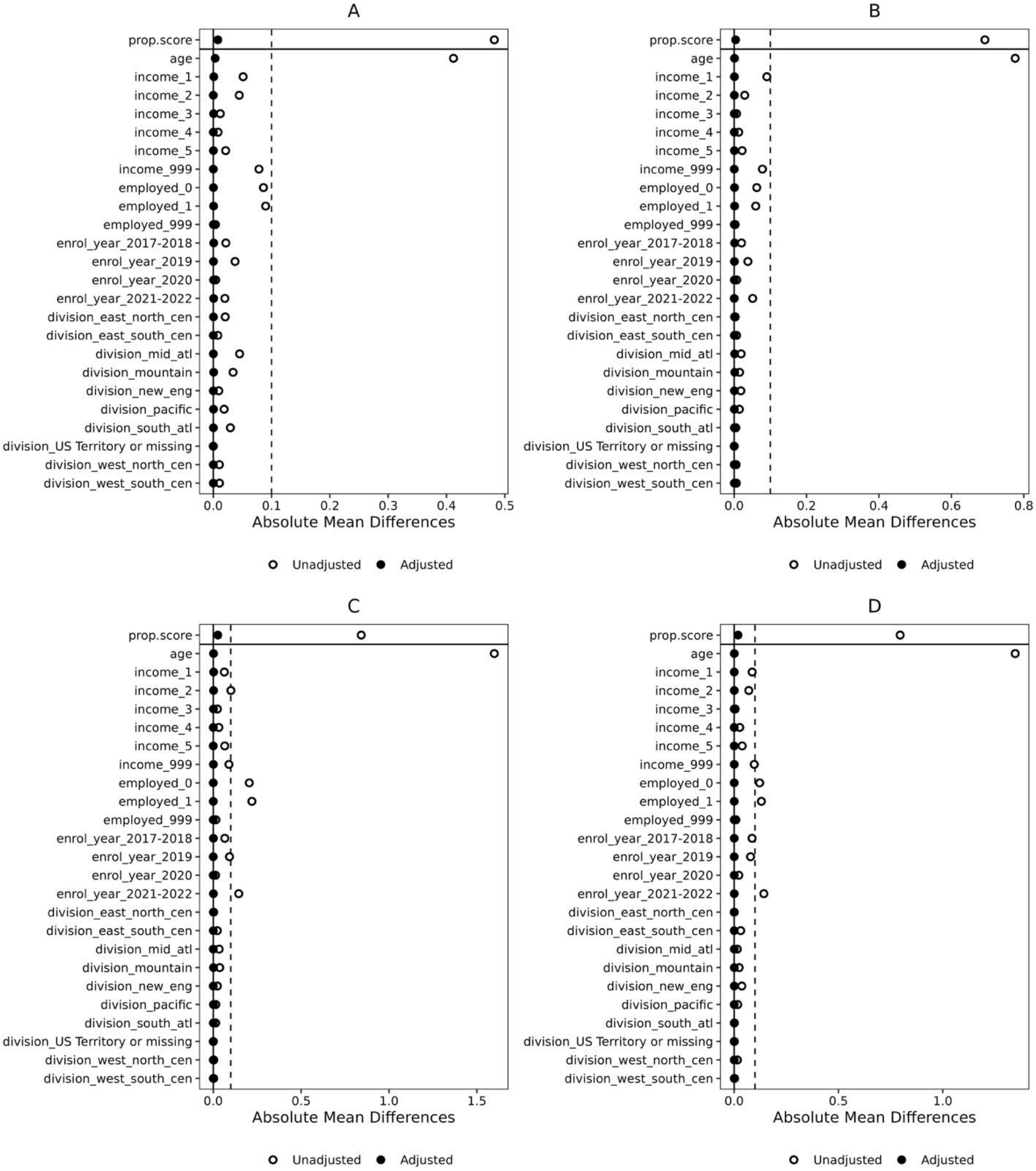

© 2023 Tran NK et al. *JAMA Network Open.*

eFigure 4. Absolute Standardized Mean Differences Between E) Gender Diverse People Assigned Male at Birth (of Any Sexual Orientation) Compared to Cisgender Heterosexual Men, F) Gender Diverse People Assigned Male at Birth (of Any Sexual Orientation) Compared to Cisgender Heterosexual Women, G) Transgender Men (of Any Sexual Orientation) Compared to Cisgender Heterosexual Men, and H) Transgender Women (of Any Sexual Orientation) Compared to Cisgender Heterosexual Women

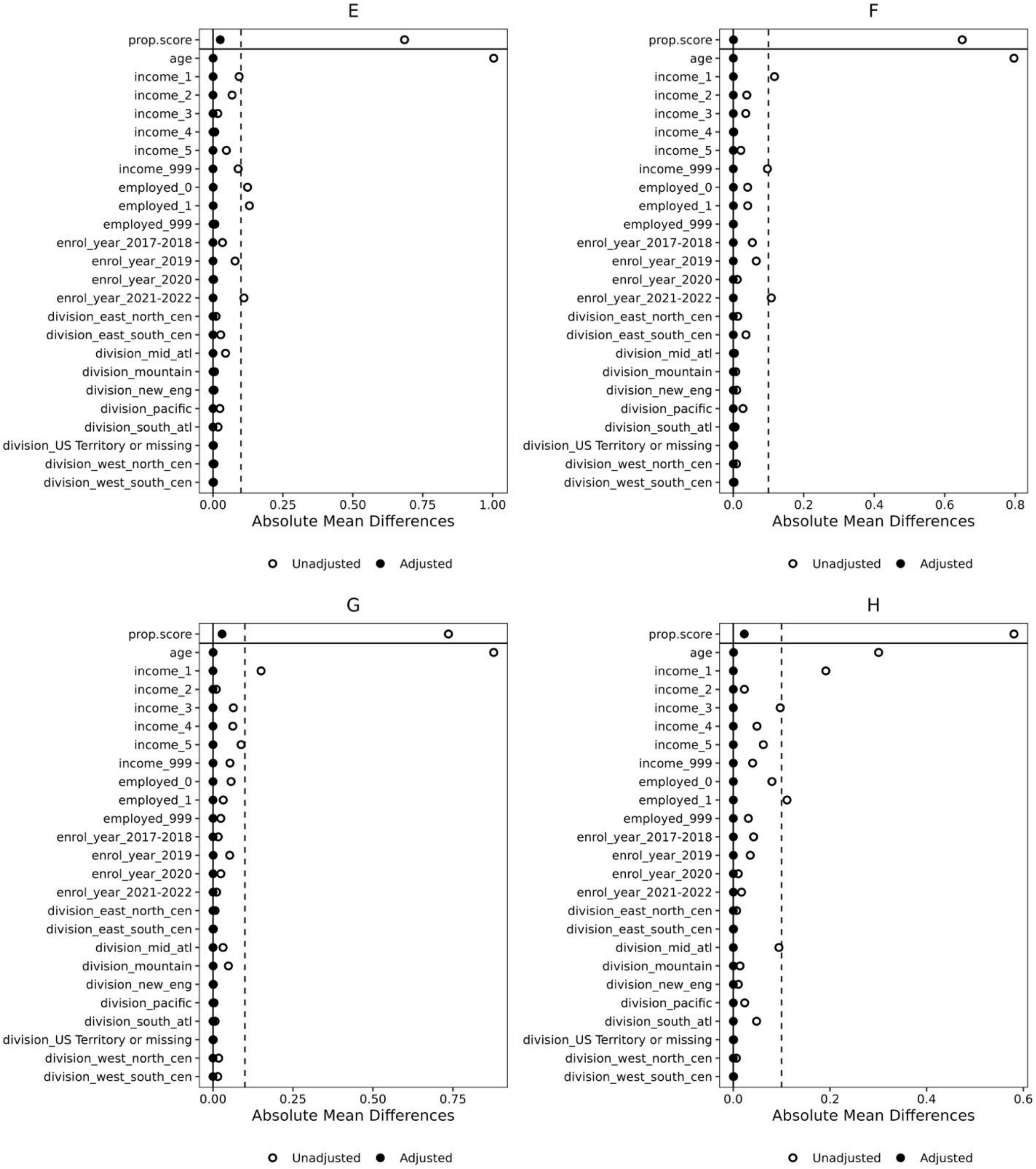

© 2023 Tran NK et al. *JAMA Network Open.*

eTable 4. Estimated Odds Ratios of 12 Health Conditions Between Cisgender Sexual Minority Men and Cisgender Heterosexual Men in the *All of Us* Research Program

Health conditions	Unadjusted OR (95% CI)	Regression adjusted[a] OR (95% CI)	PS weighted[a] OR (95% CI)
Anxiety	1.72 (1.62, 1.82)	1.71 (1.61, 1.81)	1.73 (1.63, 1.83)
Asthma	1.24 (1.14, 1.35)	1.24 (1.14, 1.35)	1.25 (1.14, 1.36)
BMI ≥ 25 kg/m^2	0.76 (0.72, 0.79)	0.82 (0.79, 0.86)	0.83 (0.79, 0.87)
Cancer	0.76 (0.71, 0.82)	1.15 (1.07, 1.23)	1.16 (1.08, 1.24)
Cardiovascular disease	0.67 (0.62, 0.72)	0.99 (0.93, 1.07)	1.01 (0.94, 1.08)
Chronic kidney disease	0.78 (0.71, 0.86)	1.06 (0.97, 1.16)	1.07 (0.97, 1.26)
Depression	1.91 (1.80, 2.02)	1.95 (1.85, 2.07)	1.98 (1.87, 2.09)
Diabetes mellitus	0.74 (0.68, 0.79)	0.91 (0.85, 0.98)	0.93 (0.87, 1.00)
HIV diagnosis	22.2 (20.3, 24.2)	21.4 (19.5, 23.4)	21.5 (19.6, 23.6)
Hypertension	0.71 (0.67, 0.75)	0.99 (0.95, 1.04)	1.01 (0.96, 1.06)
Substance use disorder	1.23 (1.14, 1.33)	1.20 (1.11, 1.29)	1.22 (1.13, 1.32)
Tobacco use disorder	1.25 (1.13, 1.37)	1.27 (1.15, 1.39)	1.30 (1.19, 1.43)

OR: Adjusted Odds Ratio; CI: Confidence Interval; BMI: Body mass index; PS: propensity score

[a] Models adjusted for current age (continuous), annual income, employment, enrollment year, and US census division.

© 2023 Tran NK et al. *JAMA Network Open.*

eTable 5. Estimated Odds Ratios of 12 Health Conditions Between Cisgender Sexual Minority Women and Cisgender Heterosexual Women in the *All of Us* Research Program

Health conditions	Unadjusted OR (95% CI)	Regression adjusted[a] OR (95% CI)	PS weighted[a] OR (95% CI)
Anxiety	1.44 (1.38, 1.51)	1.35 (1.29, 1.41)	1.42 (1.36, 1.49)
Asthma	1.28 (1.21, 1.35)	1.27 (1.21, 1.35)	1.32 (1.25, 1.39)
BMI ≥ 25 kg/m^2	0.92 (0.89, 0.96)	0.99 (0.95, 1.03)	1.05 (1.01, 1.10)
Cancer	0.50 (0.46, 0.54)	0.88 (0.81, 0.95)	0.90 (0.84, 0.97)
Cardiovascular disease	0.54 (0.49, 0.59)	0.98 (0.90, 1.07)	1.03 (0.94, 1.13)
Chronic kidney disease	0.49 (0.44, 0.56)	0.83 (0.73, 0.93)	0.86 (0.76, 0.97)
Depression	1.43 (1.36, 1.49)	1.46 (1.40, 1.53)	1.55 (1.48, 1.62)
Diabetes mellitus	0.65 (0.61, 0.70)	0.85 (0.79, 0.90)	0.91 (0.85, 0.97)
HIV diagnosis	1.37 (1.11, 1.67)	1.26 (1.02, 1.56)	1.43 (1.16, 1.76)
Hypertension	0.54 (0.51, 0.57)	0.91 (0.87, 0.95)	0.94 (0.90, 0.99)
Substance use disorder	2.41 (2.23, 2.61)	1.81(1.67, 1.96)	1.96 (1.82, 2.13)
Tobacco use disorder	1.48 (1.36, 1.60)	1.43 (1.32, 1.55)	1.60 (1.47, 1.73)

OR: Adjusted Odds Ratio; CI: Confidence Interval; BMI: Body mass index; PS: propensity score

[a] Models adjusted for current age (continuous), annual income, employment, enrollment year, and US census division.

© 2023 Tran NK et al. *JAMA Network Open.*

eTable 6. Estimated Odds Ratios of 12 Health Conditions Between Gender Diverse People Assigned Female at Birth of Any Sexual Orientation and Cisgender Heterosexual Men in the *All of Us* Research Program

Health conditions	Unadjusted OR (95% CI)	Regression adjusted[a] OR (95% CI)	PS weighted[a] OR (95% CI)
Anxiety	3.22 (2.76, 3.76)	2.97 (2.54, 3.48)	3.32 (2.84, 3.88)
Asthma	2.49 (2.03, 3.02)	2.42 (1.98, 2.96)	2.50 (2.04, 3.06)
BMI $\geq$ 25 kg/m^2	0.73 (0.63, 0.85)	0.95 (0.82, 1.11)	1.12 (0.97, 1.30)
Cancer	0.25 (0.17, 0.35)	0.91 (0.65, 1.29)	0.94 (0.67, 1.32)
Cardiovascular disease	0.19 (0.12, 0.27)	0.62 (0.42, 0.91)	0.71 (0.48, 1.03)
Chronic kidney disease	0.24 (0.14, 0.38)	0.63 (0.39, 1.02)	0.70 (0.44, 1.12)
Depression	3.12 (2.67, 3.64)	3.36 (2.87, 3.92)	3.80 (3.25, 4.44)
Diabetes mellitus	0.35 (0.26, 0.47)	0.69 (0.51, 0.92)	0.86 (0.64, 1.14)
HIV diagnosis	0.53 (0.16, 1.23)	0.62 (0.23, 1.66)	0.61 (0.19, 1.90)
Hypertension	0.28 (0.23, 0.34)	0.79 (0.66, 0.95)	0.91 (0.76, 1.10)
Substance use disorder	0.42 (0.27, 0.60)	0.35 (0.24, 0.52)	0.48 (0.33, 0.71)
Tobacco use disorder	0.66 (0.44, 0.95)	0.75 (0.51, 1.11)	1.03 (0.71, 1.51)

OR: Adjusted Odds Ratio; CI: Confidence Interval; BMI: Body mass index; PS: propensity score

[a] Models adjusted for current age (continuous), annual income, employment, enrollment year, and US census division.

eTable 7. Estimated Odds Ratios of 12 Health Conditions Between Gender Diverse People Assigned Female at Birth of Any Sexual Orientation and Cisgender Heterosexual Women in the *All of Us* Research Program

Health conditions	Unadjusted OR (95% CI)	Regression adjusted[a] OR (95% CI)	PS weighted[a] OR (95% CI)
Anxiety	1.90 (1.63, 2.22)	1.75 (1.51, 2.05)	1.93 (1.66, 2.25)
Asthma	1.27 (1.04, 1.54)	1.29 (1.06, 1.56)	1.40 (1.15, 1.70)
BMI ≥ 25 kg/m^2	0.78 (0.68, 0.91)	0.89 (0.77, 1.03)	1.06 (0.92, 1.23)
Cancer	0.30 (0.20, 0.43)	0.71 (0.50, 1.02)	0.78 (0.44, 1.11)
Cardiovascular disease	0.40 (0.26, 0.59)	0.99 (0.67, 1.45)	1.07 (0.73, 1.57)
Chronic kidney disease	0.41 (0.24, 0.65)	0.92 (0.58, 1.48)	1.00 (0.62, 1.60)
Depression	1.92 (1.64, 2.23)	2.01 (1.72, 2.34)	2.26 (1.94, 2.62)
Diabetes mellitus	0.40 (0.29, 0.53)	0.62 (0.46, 0.82)	0.73 (0.55, 0.97)
HIV diagnosis	0.76 (0.24, 1.79)	0.58 (0.19, 1.83)	0.75 (0.24, 2.36)
Hypertension	0.35 (0.28, 0.43)	0.78 (0.64, 0.94)	0.87 (0.72, 1.04)
Substance use disorder	1.11 (0.74, 1.60)	0.83 (0.56, 1.23)	1.05 (0.72, 1.54)
Tobacco use disorder	0.83 (0.55, 1.20)	0.83 (0.56, 1.21)	1.07 (0.73, 1.57)

OR: Adjusted Odds Ratio; CI: Confidence Interval; BMI: Body mass index; PS: propensity score

[a] Models adjusted for current age (continuous), annual income, employment, enrollment year, and US census division.

© 2023 Tran NK et al. *JAMA Network Open.*

eTable 8. Estimated Odds Ratios of 12 Health Conditions Between Gender Diverse People Assigned Male at Birth of Any Sexual Orientation and Cisgender Heterosexual Men in the *All of Us* Research Program

Health conditions	Unadjusted OR (95% CI)	Regression adjusted[a] OR (95% CI)	PS weighted[a] OR (95% CI)
Anxiety	2.20 (1.69, 2.84)	2.03 (1.57, 2.62)	2.19 (1.70, 2.83)
Asthma	0.77 (0.45, 1.22)	0.74 (0.45, 1.21)	0.76 (0.46, 1.24)
BMI ≥ 25 kg/m^2	0.50 (0.40, 0.62)	0.61 (0.49, 0.75)	0.67 (0.54, 0.83)
Cancer	0.43 (0.27, 0.66)	1.15 (0.77, 1.71)	1.13 (0.75, 1.69)
Cardiovascular disease	0.33 (0.20, 0.53)	0.83 (0.52, 1.32)	0.86 (0.53, 1.38)
Chronic kidney disease	0.18 (0.06, 0.39)	0.37 (0.15, 0.89)	0.38 (0.16, 0.92)
Depression	1.98 (1.51, 2.56)	2.06 (1.58, 2.68)	2.24 (1.72, 2.92)
Diabetes mellitus	0.44 (0.28, 0.65)	0.69 (0.45, 1.05)	0.76 (0.50, 1.17)
HIV diagnosis	3.67 (1.88, 6.39)	4.06 (2.23, 7.40)	4.63 (2.54, 8.44)
Hypertension	0.32 (0.23, 0.43)	0.69 (0.52, 0.92)	0.74 (0.55, 0.99)
Substance use disorder	0.91 (0.58, 1.36)	0.75 (0.49, 1.13)	0.89 (0.60, 1.33)
Tobacco use disorder	0.72 (0.38, 1.23)	0.79 (0.45, 1.39)	0.96 (0.55, 1.68)

OR: Adjusted Odds Ratio; CI: Confidence Interval; BMI: Body mass index; PS: propensity score

[a] Models adjusted for current age (continuous), annual income, employment, enrollment year, and US census division.

© 2023 Tran NK et al. *JAMA Network Open.*

eTable 9. Estimated Odds Ratios of 12 Health Conditions Between Gender Diverse People Assigned Male at Birth of Any Sexual Orientation and Cisgender Heterosexual Women in the *All of Us* Research Program

**Health conditions**	**Unadjusted OR (95% CI)**	**Regression adjusted[a] OR (95% CI)**	**PS weighted[a] OR (95% CI)**
Anxiety	1.30 (1.00, 1.68)	1.21 (0.94, 1.56)	1.29 (1.00, 1.66)
Asthma	0.39 (0.23, 0.62)	0.39 (0.24, 0.63)	0.41 (0.25, 0.66)
BMI ≥ 25 kg/m^2	0.53 (0.43, 0.66)	0.58 (0.47, 0.73)	0.64 (0.51, 0.79)
Cancer	0.52 (0.32, 0.80)	0.98 (0.65, 1.48)	1.01 (0.67, 1.52)
Cardiovascular disease	0.71 (0.42, 1.12)	1.32 (0.82, 2.12)	1.37 (0.85, 2.20)
Chronic kidney disease	0.31 (0.11, 0.68)	0.54 (0.22, 1.30)	0.56 (0.23, 1.35)
Depression	1.21 (0.93, 1.57)	1.24 (0.96, 1.62)	1.35 (1.04, 1.75)
Diabetes mellitus	0.50 (0.31, 0.74)	0.63 (0.41, 0.96)	0.68 (0.45, 1.03)
HIV diagnosis	5.31 (2.72, 9.25)	5.00 (2.74, 9.13)	5.61 (3.09, 10.2)
Hypertension	0.40 (0.28, 0.54)	0.69 (0.52, 0.92)	0.72 (0.54, 0.96)
Substance use disorder	2.42 (1.54, 3.63)	1.76 (1.15, 2.67)	1.98 (1.32, 2.97)
Tobacco use disorder	0.91 (0.48, 1.55)	0.87 (0.49, 1.53)	1.00 (0.57, 1.74)

OR: Adjusted Odds Ratio; CI: Confidence Interval; BMI: Body mass index; PS: propensity score

[a] Models adjusted for current age (continuous), annual income, employment, enrollment year, and US census division.

© 2023 Tran NK et al. *JAMA Network Open.*

eTable 10. Estimated Odds Ratios of 12 Health Conditions Between Transgender Men of Any Sexual Orientation and Cisgender Heterosexual Men in the *All of Us* Research Program

Health conditions	Unadjusted OR (95% CI)	Regression adjusted[a] OR (95% CI)	PS weighted[a] OR (95% CI)
Anxiety	2.08 (1.72, 2.51)	1.96 (1.62, 2.35)	2.08 (1.73, 2.49)
Asthma	1.89 (1.47, 2.39)	1.87 (1.47, 2.38)	1.88 (1.47, 2.39)
BMI ≥ 25 kg/m^2	1.26 (1.06, 1.50)	1.65 (1.38, 1.96)	1.68 (1.41, 2.00)
Cancer	0.42 (0.29, 0.57)	0.98 (0.73, 1.33)	0.99 (0.73, 1.35)
Cardiovascular disease	0.36 (0.25, 0.49)	0.68 (0.49, 0.94)	0.69 (0.49, 0.95)
Chronic kidney disease	0.57 (0.39, 0.81)	0.87 (0.61, 1.25)	0.88 (0.61, 1.27)
Depression	2.18 (1.80, 2.61)	2.16 (1.80, 2.59)	2.29 (1.91, 2.74)
Diabetes mellitus	0.86 (0.68, 1.08)	1.17 (0.94, 1.46)	1.24 (1.00, 1.55)
HIV diagnosis	2.16 (1.18, 3.60)	1.80 (1.05, 3.06)	1.97 (1.16, 3.35)
Hypertension	0.58 (0.48, 0.70)	1.06 (0.89, 1.25)	1.09 (0.92, 1.29)
Substance use disorder	0.98 (0.72, 1.30)	0.65 (0.49, 0.87)	0.70 (0.53, 0.94)
Tobacco use disorder	1.06 (0.73, 1.47)	0.97 (0.69, 1.37)	1.06 (0.76, 1.49)

OR: Adjusted Odds Ratio; CI: Confidence Interval; BMI: Body mass index; PS: propensity score

[a] Models adjusted for current age (continuous), annual income, employment, enrollment year, and US census division.

© 2023 Tran NK et al. *JAMA Network Open.*

eTable 11. Estimated Odds Ratios of 12 Health Conditions Between Transgender Women of Any Sexual Orientation and Cisgender Heterosexual Women in the *All of Us* Research Program

Health conditions	Unadjusted OR (95% CI)	Regression adjusted[a] OR (95% CI)	PS weighted[a] OR (95% CI)
Anxiety	0.94 (0.76, 1.14)	0.90 (0.74, 1.10)	0.92 (0.75, 1.12)
Asthma	0.53 (0.39, 0.71)	0.51 (0.38, 0.69)	0.52 (0.38, 0.70)
BMI $\geq$ 25 kg/m^2	0.91 (0.77, 1.07)	0.82 (0.70, 0.96)	0.83 (0.81, 0.97)
Cancer	0.56 (0.41, 0.76)	0.78 (0.58, 1.05)	0.79 (0.59, 1.06)
Cardiovascular disease	1.00 (0.73, 1.34)	1.10 (0.83, 1.46)	1.12 (0.84, 1.49)
Chronic kidney disease	1.07 (0.74, 1.50)	1.11 (0.79, 1.57)	1.12 (0.79, 1.57)
Depression	1.09 (0.90, 1.32)	1.03 (0.85, 1.25)	1.06 (0.87, 1.28)
Diabetes mellitus	0.95 (0.74, 1.19)	0.87 (0.69, 1.09)	0.89 (0.71, 1.11)
HIV diagnosis	10.8 (7.74, 14.6)	5.84 (4.22, 8.09)	6.01 (4.35, 8.30)
Hypertension	0.85 (0.71, 1.02)	0.95 (0.80, 1.11)	0.96 (0.81, 1.12)
Substance use disorder	3.07 (2.31, 4.00)	2.02 (1.54, 2.65)	2.06 (1.57, 2.71)
Tobacco use disorder	1.56 (1.11, 2.12)	1.27 (0.92, 1.75)	1.31 (0.94, 1.80)

OR: Adjusted Odds Ratio; CI: Confidence Interval; BMI: Body mass index; PS: propensity score

[a] Models adjusted for current age (continuous), annual income, employment, enrollment year, and US census division.
