# Supplementary material for: Prevalence of 12 Common Health Conditions in Sexual and Gender Minority Participants in the All of Us Research Program
Source: JAMA Netw Open. 2023 Jul 31;6(7):e2324969. doi: 10.1001/jamanetworkopen.2023.24969 (PMC10391317; doi:10.1001/jamanetworkopen.2023.24969)
Supplement: Supplement 2. — Data Sharing Statement [file jamanetwopen-e2324969-s002.pdf]

Data Sharing Statement

Tran. Prevalence of 12 Common Health Conditions in Sexual and Gender Minority Participants in the All of Us Research Program. *JAMA Netw Open*. Published July 31, 2023. doi:10.1001/jamanetworkopen.2023.24969

Data
Data available: Yes
Data types: Deidentified participant data
How to access data: Analytic datasets are available in the All of Us Researcher Workbench for researchers with controlled tier access: researchallofus.org
When available: With publication

Supporting Documents
Document types: Statistical/analytic code
How to access documents: Relevant R code will be made publicly available through the All of Us Research Workbench: researchallofus.org
When available: With publication

Additional Information
Who can access the data: Data will be available to investigators with control tier access through All of Us Research Workbench.
Types of analyses: Analyses are available for replication purposes.
Mechanisms of data availability: Data will be made available to those after a data use agreement between All of Us and their institution has been approved. This will grant researchers controlled tier access to the analytic data.
